# Supplementary material for: Impact of point-of-care tests in community pharmacies: a systematic review and meta-analysis
Source: BMJ Open. 2020 May 15;10(5):e034298. doi: 10.1136/bmjopen-2019-034298 (PMC7232628; doi:10.1136/bmjopen-2019-034298)
Supplement: Supplementary data [file bmjopen-2019-034298supp006.pdf]

# ▲	Searches
1	((rapid$ or same time or same visit or near patient or portable or handheld or hand-held) adj3 (test$ or analys$ or analyz$ or measure$ or assay$ or monitor* or device*)).ti,ab.
2	(fingerprick or finger prick).tw.
3	(poc or poct or "point of care").tw.
4	point-of-care systems/ or point-of-care testing/
5	1 or 2 or 3 or 4
6	Community Pharmacy Services/
7	Pharmacists/
8	(pharmacy or pharmacies or pharmacist?).ti,ab.
9	6 or 7 or 8
10	5 and 9
11	randomized controlled trial.pt.
12	controlled clinical trial.pt.
13	randomized.ab.
14	placebo.ab.
15	drug therapy.fs.
16	randomly.ab.
17	trial.ab.
18	groups.ab.
19	11 or 12 or 13 or 14 or 15 or 16 or 17 or 18
20	(animals not (humans and animals)).sh.
21	19 not 20
22	10 and 21
23	10 not 22

Supplementary Table 1: Medline search strategy
