# Supplementary material for: Impact of point-of-care tests in community pharmacies: a systematic review and meta-analysis
Source: BMJ Open. 2020 May 15;10(5):e034298. doi: 10.1136/bmjopen-2019-034298 (PMC7232628; doi:10.1136/bmjopen-2019-034298)

Supplementary Figure 1 - Risk of bias for pre-post observational studies

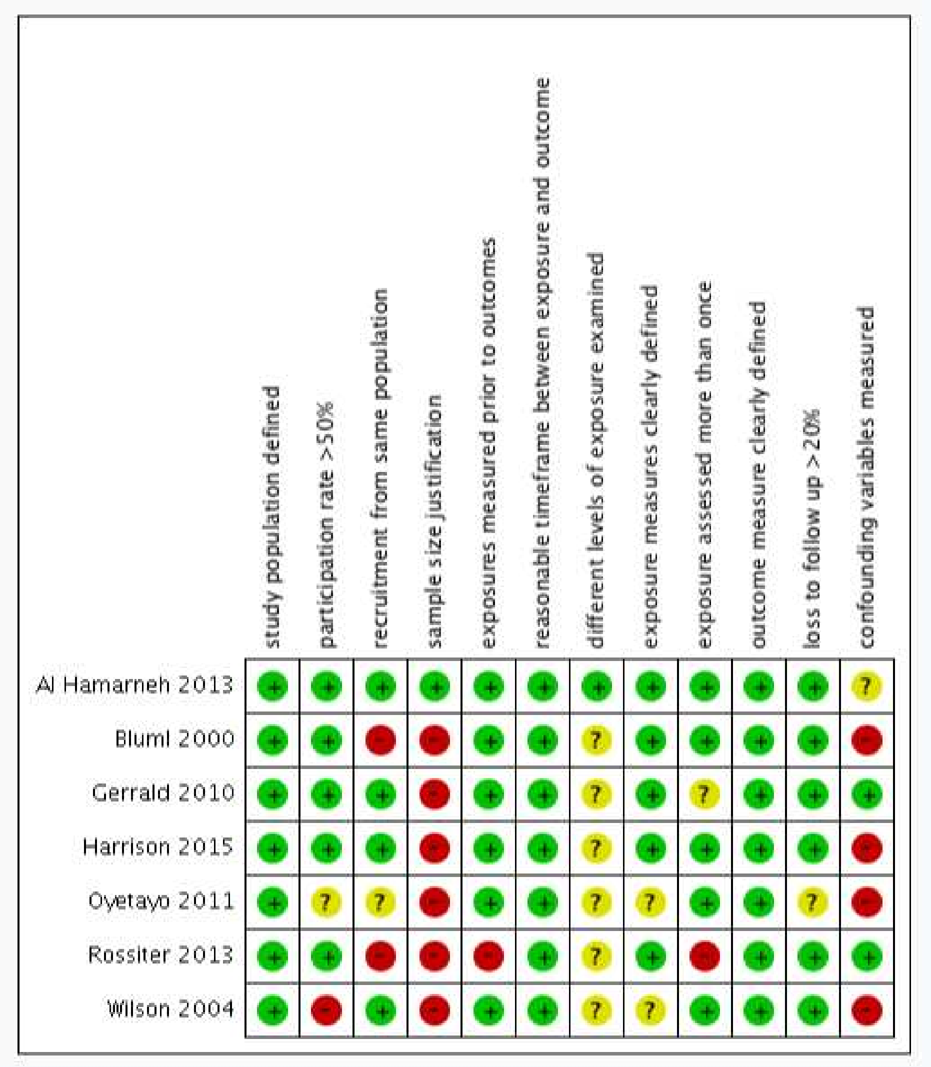

Supplement: Supplementary data [file bmjopen-2019-034298supp001.pdf]
