# Supplementary material for: Impact of point-of-care tests in community pharmacies: a systematic review and meta-analysis
Source: BMJ Open. 2020 May 15;10(5):e034298. doi: 10.1136/bmjopen-2019-034298 (PMC7232628; doi:10.1136/bmjopen-2019-034298)
Supplement: Supplementary data [file bmjopen-2019-034298supp003.pdf]

Supplementary Figure 3 - Risk of bias for prospective controlled trials

	Random sequence generation (selection bias)	Allocation concealment (selection bias)	Blinding of participants and personnel (performance bias)	Blinding of outcome assessment (detection bias)	Incomplete outcome data (attrition bias)	Selective reporting (reporting bias)	Other bias
Ansah 2015	+	-	-	+	+	+	+
Deepalakshmi 2018	-	-	-	-	+	?	+
Ikwuobe 2013	+	-	-	-	?	?	+
Mbonye 2015	+	-	-	+	+	+	+
Peterson 2004	+	-	-	-	?	?	+
