# Supplementary material for: Impact of point-of-care tests in community pharmacies: a systematic review and meta-analysis
Source: BMJ Open. 2020 May 15;10(5):e034298. doi: 10.1136/bmjopen-2019-034298 (PMC7232628; doi:10.1136/bmjopen-2019-034298)
Supplement: Supplementary data [file bmjopen-2019-034298supp004.pdf]

Study or Subgroup	point-of-care Mean [mg/dL]	point-of-care SD [mg/dL]	point-of-care Total	control Mean [mg/dL]	control SD [mg/dL]	control Total	Weight	Mean Difference IV, Random, 95% CI [mg/dL]
**3.5.1 total cholesterol randomised controlled trial**								
Peterson 2004	170.1	23.2	39	177.9	30.9	42	100.0%	-7.80 [-19.65, 4.05]
**Subtotal (95% CI)**			**39**			**42**	**100.0%**	**-7.80 [-19.65, 4.05]**
Heterogeneity: Not applicable								
Test for overall effect: Z = 1.29 (P = 0.20)								
**3.5.3 total cholesterol pre-post observational**								
Bluml 2000	207.5	41.1	396	238	46.7	396	85.1%	-30.50 [-36.63, -24.37]
Gerrald 2010	150.2	34.6	81	174.9	57.6	81	14.9%	-24.70 [-39.33, -10.07]
**Subtotal (95% CI)**			**477**			**477**	**100.0%**	**-29.63 [-35.29, -23.98]**
Heterogeneity: $Tau^2 = 0.00$; $Chi^2 = 0.51$, df = 1 (P = 0.47); $I^2 = 0\%$								
Test for overall effect: Z = 10.28 (P < 0.00001)								
**3.5.4 LDL-cholesterol**								
Bluml 2000	119.8	35.7	387	153.7	46.7	387	59.3%	-33.90 [-39.76, -28.04]
Gerrald 2010	81.5	28.1	76	103.1	45.1	76	40.7%	-21.60 [-33.55, -9.65]
**Subtotal (95% CI)**			**463**			**463**	**100.0%**	**-28.90 [-40.74, -17.06]**
Heterogeneity: $Tau^2 = 52.60$; $Chi^2 = 3.28$, df = 1 (P = 0.07); $I^2 = 70\%$								
Test for overall effect: Z = 4.78 (P < 0.00001)								
**3.5.5 HDL-cholesterol**								
Bluml 2000	49.2	16.5	394	43.1	14.1	394	56.3%	6.10 [3.96, 8.24]
Gerrald 2010	39.6	13.1	81	38.4	13.1	81	43.7%	1.20 [-2.83, 5.23]
**Subtotal (95% CI)**			**475**			**475**	**100.0%**	**3.96 [-0.80, 8.72]**
Heterogeneity: $Tau^2 = 9.29$; $Chi^2 = 4.42$, df = 1 (P = 0.04); $I^2 = 77\%$								
Test for overall effect: Z = 1.63 (P = 0.10)								
**3.5.6 triglycerides**								
Bluml 2000	195	91.3	394	216.6	111.3	394	84.5%	-21.60 [-35.81, -7.39]
Gerrald 2010	141.4	84.8	81	163.5	126.7	81	15.5%	-22.10 [-55.30, 11.10]
**Subtotal (95% CI)**			**475**			**475**	**100.0%**	**-21.68 [-34.74, -8.61]**
Heterogeneity: $Tau^2 = 0.00$; $Chi^2 = 0.00$, df = 1 (P = 0.98); $I^2 = 0\%$								
Test for overall effect: Z = 3.25 (P = 0.001)								

Test for subgroup differences: $Chi^2 = 90.94$, df = 4 (P < 0.00001), $I^2 = 95.6\%$

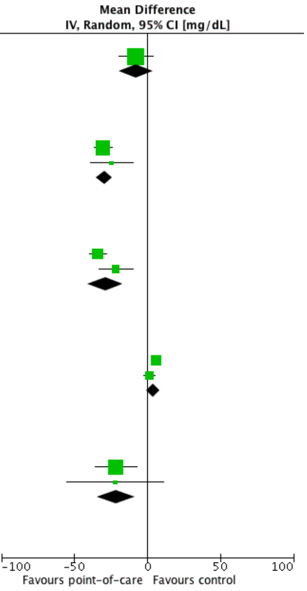

Supplementary Figure 4 – The effect of pharmacy point-of-care-testing on lipid control - Total cholesterol, LDL cholesterol, HDL cholesterol, and Triglycerides (mg/dL)
