# Supplementary material for: Impact of point-of-care tests in community pharmacies: a systematic review and meta-analysis
Source: BMJ Open. 2020 May 15;10(5):e034298. doi: 10.1136/bmjopen-2019-034298 (PMC7232628; doi:10.1136/bmjopen-2019-034298)
Supplement: Supplementary data [file bmjopen-2019-034298supp005.pdf]

Study or Subgroup	point-of-care Mean [%]	point-of-care SD [%]	point-of-care Total	control Mean [%]	control SD [%]	control Total	Weight	Mean Difference IV, Random, 95% CI [%]
Al Hamarneh 2013	7.3	0.9	93	9.1	1	100	51.5%	−1.80 [−2.07, −1.53]
Oyetayo 2011	7.6	2.42	126	7.8	2.42	126	48.5%	−0.20 [−0.80, 0.40]
**Total (95% CI)**			**219**			**226**	**100.0%**	**−1.02 [−2.59, 0.54]**

Heterogeneity: $Tau^2 = 1.22$; $Chi^2 = 22.93$, $df = 1$ ($P < 0.00001$); $I^2 = 96\%$
Test for overall effect: $Z = 1.28$ ($P = 0.20$)

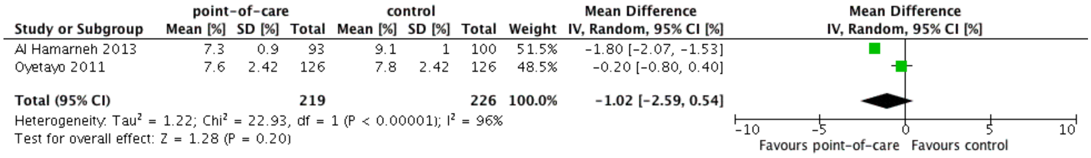

Supplementary Figure 5 – The effect of pharmacy point-of-care-testing on glycated haemoglobin HbA1c control (%)
