# Supplementary material for: Impact of point-of-care tests in community pharmacies: a systematic review and meta-analysis
Source: BMJ Open. 2020 May 15;10(5):e034298. doi: 10.1136/bmjopen-2019-034298 (PMC7232628; doi:10.1136/bmjopen-2019-034298)
Supplement: Author's manuscript [file bmjopen-2019-034298.draft_revisions.pdf]

BMJ Open is committed to open peer review. As part of this commitment we make the peer review history of every article we publish publicly available.

When an article is published we post the peer reviewers' comments and the authors' responses online. We also post the versions of the paper that were used during peer review. These are the versions that the peer review comments apply to.

The versions of the paper that follow are the versions that were submitted during the peer review process. They are not the versions of record or the final published versions. They should not be cited or distributed as the published version of this manuscript.

BMJ Open is an open access journal and the full, final, typeset and author-corrected version of record of the manuscript is available on our site with no access controls, subscription charges or pay-per-view fees (http://bmjopen.bmj.com).

If you have any questions on BMJ Open's open peer review process please email info.bmjopen@bmj.com

BMJ Open

Impact of point-of-care tests by community pharmacists: a systematic review and meta-analysis

Journal:	*BMJ Open*
Manuscript ID	bmjopen-2019-034298
Article Type:	Original research
Date Submitted by the Author:	13-Sep-2019

Complete List of Authors:	Albasri, Ali; University of Oxford , Nuffield Department of Primary Care Health Sciences<br>Van Den Bruel, Ann; KU Leuven Department of Public Health and Primary Care<br>Hayward, Gail; University of Oxford, Nuffield Department of Primary Care Health Sciences<br>McManus, Richard; University of Oxford, Dept of Primary Care Health Sciences<br>Sheppard, James; University of Oxford, Nuffield Department of Primary Care Health Sciences<br>Verbakel, Jan; KU Leuven (University of Leuven), Department of Primary Care; University of Oxford, Nuffield Department of Primary Care Health Sciences

Keywords:	point of care testing, community pharmacy, Organisation of health services < HEALTH SERVICES ADMINISTRATION & MANAGEMENT

SCHOLARONE™
Manuscripts

I, the Submitting Author has the right to grant and does grant on behalf of all authors of the Work (as defined in the below author licence), an exclusive licence and/or a non-exclusive licence for contributions from authors who are: i) UK Crown employees; ii) where BMJ has agreed a CC-BY licence shall apply, and/or iii) in accordance with the terms applicable for US Federal Government officers or employees acting as part of their official duties; on a worldwide, perpetual, irrevocable, royalty-free basis to BMJ Publishing Group Ltd ("BMJ") its licensees and where the relevant Journal is co-owned by BMJ to the co-owners of the Journal, to publish the Work in this journal and any other BMJ products and to exploit all rights, as set out in our licence.

The Submitting Author accepts and understands that any supply made under these terms is made by BMJ to the Submitting Author unless you are acting as an employee on behalf of your employer or a postgraduate student of an affiliated institution which is paying any applicable article publishing charge ("APC") for Open Access articles. Where the Submitting Author wishes to make the Work available on an Open Access basis (and intends to pay the relevant APC), the terms of reuse of such Open Access shall be governed by a Creative Commons licence – details of these licences and which Creative Commons licence will apply to this Work are set out in our licence referred to above.

Other than as permitted in any relevant BMJ Author's Self Archiving Policies, I confirm this Work has not been accepted for publication elsewhere, is not being considered for publication elsewhere and does not duplicate material already published. I confirm all authors consent to publication of this Work and authorise the granting of this licence.

For peer review only

Impact of point-of-care tests by community pharmacists: a systematic review and meta-analysis

Ali Albasri[1], Ann Van den Bruel[2], Gail Hayward[1], Richard J McManus[1], James P Sheppard[1], Jan Y Verbakel[1*]

[1]Nuffield Department of Primary Care Health Sciences, University of Oxford, Woodstock Road, OX26GG, Oxford, Oxfordshire, United Kingdom

2 Academic Centre for Primary Care, KU Leuven, Kapucijnenvoer 33 blok J, 3000 Leuven, Belgium

Email addresses:

ali.albasri@phc.ox.ac.uk, ann.vandenbruel@kuleuven.be, gail.hayward@phc.ox.ac.uk, richard.mcmanus@phc.ox.ac.uk, james.sheppard@phc.ox.ac.uk, jan.verbakel@phc.ox.ac.uk

[*]Corresponding author: Jan Y Verbakel, Nuffield Department of Primary Care Health Sciences, Radcliffe Primary Care Building, Woodstock Road, Oxford OX2 6GG, UK jan.verbakel@phc.ox.ac.uk

For peer review only

For peer review only - http://bmjopen.bmj.com/site/about/guidelines.xhtml

Abstract

Background

Point of care tests (POCTs) have been increasingly proposed as clinical tools to aid diagnosis of acute conditions as well as for the monitoring of clinical parameters in chronic conditions. The pharmacy setting has become increasingly involved in the use of POCT in recent years. This systematic review aimed to summarise the literature regarding the use of POCT in pharmacies versus control/usual care.

Method

Five databases were searched for articles that: involved a POCT conducted by a community pharmacist, member of pharmacy staff or local equivalent; measured a clinically relevant outcome e.g. clinical parameter monitoring. No clinical condition, study design or language limits were set. Data were combined using random-effects meta-analyses.

Results

Searches generated 1,584 unique articles, 13 of which were included in the meta-analyses. The included studies covered four main therapeutic areas: targeted anti-malarial therapy (n=3 studies), HbA1c in diabetes (n= 2 studies), lipid control (n=3 studies), and INR control in patients taking Warfarin (n=5 studies). POCT in pharmacies reduced the risk of receiving antimalarial treatment when not clinically indicated (RR 0.34 95% CI 0.31-0.37). Lipid and HbA1c control appeared largely unaffected by pharmacy POCTs, and the impact on INR time-in-therapeutic-range was inconclusive.

Conclusion

Only 4/13 included studies were randomised controlled trials (RCTs) and none were conducted in the UK, limiting our ability to conclusively determine the clinical utility of POCT conducted in pharmacies. Further RCTs are needed, particularly in areas such as upper respiratory tract infections, which have gathered momentum among UK service commissioners in recent years.

For peer review only

For peer review only - http://bmjopen.bmj.com/site/about/guidelines.xhtml

Strengths and limitations of this study:

- A timely and comprehensive review of POCT conducted in pharmacies vs a control/comparator.
- We identified gaps in the literature regarding the evidence for use of POCT in pharmacies, particularly in areas such as the triage and treatment of common acute bacterial or viral respiratory tract infections, where no evidence was found.
- Studies where the use of POCTs were embedded among other interventions were not included in this review. Such studies may also provide useful information regarding the contribution of POCT to clinical outcomes.

For peer review only

For peer review only - http://bmjopen.bmj.com/site/about/guidelines.xhtml

Introduction

A point-of-care test (POCT) can be defined as a test performed by a qualified member of staff nearby the patient, where results are made available within the same clinical visit to support clinical decision making.(1) These tests have the potential to save clinical time and improve patient access to care in the form of diagnoses, medications or dose amendments.(2)

Interest in the use of POCTs in different healthcare settings is increasing and is expected to grow significantly in the years to come.(3) In 2016, the 'Community pharmacy forward view' (CPFV) was published as a response by the pharmacy sector to the then 'NHS five year forward view', and suggested that diagnostics and POCTs should be made routinely available in pharmacy settings.(4)

Given the current strain on primary healthcare services,(5) the provision of POCTs has become more commonplace in UK community pharmacies, with particular emphasis on the potential for POCTs to aid both acute condition diagnosis and long-term condition management.(6) In 2016, NHS England approved a 'test and treat' service at a large pharmacy chain for patients presenting with sore throats, in an attempt to curb inappropriate antibiotic prescriptions and reduce burden on general practice.(7) However, the evidence behind the use of POCTs in pharmacies appears to be from either pilot studies, non-randomised studies, or studies with no comparator groups.(8) The evidence-base for implementing POCTs remains a concern more generally given that studies tend to focus on test performance (method comparison with central laboratory testing) rather than clinical or healthcare utilization outcomes.(9)

While previous work has focused on the analytical quality of POCTs used by community pharmacists,(10) this paper presents the findings of a systematic review and meta-analysis assessing the clinical impact of POCTs in community pharmacies on clinical outcomes and healthcare processes.

For peer review only - http://bmjopen.bmj.com/site/about/guidelines.xhtml

Methods

Search strategy

A comprehensive search strategy in MEDLINE, Cochrane Central Register of Controlled Trials, EMBASE, ClinicalTrial.gov and Web of Science was devised. An example of the MEDLINE search terms can be found in supplementary file 1 (online). Relevant articles from inception to 24/04/2019 were searched in addition to references of relevant reviews and articles that met our selection criteria. No language limits or study design filters were applied.

Selection of studies and inclusion criteria

Two members of the review team (AA and JYV) independently reviewed titles, abstracts and full texts. Studies screened by title, abstract and full text were eligible for inclusion if they met all of the following criteria:

1. A POCT conducted by a community pharmacist or member of community pharmacy staff (i.e. pharmacy technician, healthcare assistant, or local equivalent).
2. Clinically relevant outcome measures reported e.g. change in clinical care such as: referral, admission to hospital, morbidity, mortality, or rate of diagnosis, time in therapeutic range, duration of illness.
3. Patients of all ages presenting to a community pharmacy for any medical condition.

Randomised controlled trials, non-randomised but experimental and controlled studies including before-and-after and case-controlled studies were included in this review. Systematic reviews were excluded but their reference lists were searched for relevant primary studies.

Studies were excluded if any of the following criteria applied:

1. Were diagnostic accuracy studies (focusing only on the performance of one or more point-of-care tests versus a central lab test).
2. Included only hospital inpatients.
3. Studies without a control group or comparator.
4. Patients self-testing or tests that were taken away by patients (to test at home, for example).

Outcomes measured

For peer review only - http://bmjopen.bmj.com/site/about/guidelines.xhtml

The primary outcome of this review was the impact of POCT on clinically relevant outcomes such as changes to treatment, disease marker monitoring, referrals, admissions to hospital, morbidity, mortality, time to diagnosis, time in therapeutic range, or duration of illness.

Data extraction

Data were independently extracted and verified by two members of the review team (AA and JYV). Data were extracted to capture changes in clinical care that resulted from the use of the POCTs. The following data were extracted from the primary studies where available: referral or admission to other healthcare providers, mortality, morbidity, time in therapeutic range, percentage of patients reaching therapeutic targets such as cholesterol and HbA1c, resulting medication recommendations, or appropriateness of medication recommendations.

Quality assessment

The methodological quality of included studies was assessed independently by two authors (AA and JYV). Randomised trials were assessed using the Cochrane Risk of Bias tool (11) and included analysis of randomisation, allocation concealment, comparison of baseline characteristics and blinding. For non-randomised but experimental and controlled studies, the Cochrane Risk of Bias tool for observational studies was used. Case-control studies were assessed using the Newcastle-Ottawa scale.(12)

Data synthesis

Meta-analyses were conducted separately for randomised controlled trials and non-randomised studies. Data were analysed using a random-effects model due to expected heterogeneity in study designs and populations.(13) Analyses were grouped according to the condition to which the POCT related. Data were combined using the Review Manager (RevMan) version 5 software. For outcomes where meta-analysis was not possible, results were described qualitatively.

Where statistical heterogeneity was detected, possible contributing factors such as the setting or operator, the patient population, and/or other methodological characteristics were investigated in sensitivity analyses where possible. Where data allowed, publication bias was assessed via Egger's test to check for small study effects.(14)

For peer review only - http://bmjopen.bmj.com/site/about/guidelines.xhtml

Data are presented as a proportion of each study population, means with standard deviation (SD) or 95% confidence intervals (CI) unless otherwise stated.

The study protocol was registered on PROSPERO: International Prospective Register of Systematic Reviews and can be found online (http://www.crd.york.ac.uk/prospero) – registration number [CRD42017048578]. This review is reported according to the PRISMA checklist for reporting systematic reviews (see supplementary file).

For peer review only

For peer review only - http://bmjopen.bmj.com/site/about/guidelines.xhtml

Results

Study selection

After removal of 619 duplicate records, 1,584 studies were identified from the literature searches and one additional record was found from citation searches. After title and abstract screening 1,513 studies were excluded leaving 71 studies to be screened by full text. Of these, 58 studies were excluded for the reasons stated in the PRISMA flow diagram (Figure 1) leaving 13 studies eligible for inclusion.

Study characteristics

Table 1 shows the characteristics of included studies: seven were observational (pre/post design),(15–21) four were randomised controlled trials,(22–25), one a prospective controlled staggered parallel design study, and one a retrospective case/control study.(26) Studies were conducted in the USA (n=5), Canada (n=2), Australia (n=1), New Zealand (n=1), Ghana (n=1), Nigeria (n=1), India (n=1), Uganda (n=1) and included data from a total of 23,149 patients. All point of care tests were conducted by a community pharmacist(s) or local equivalent that received training in both delivering the POCT and in the subsequent treatment recommendation.

None of the seven observational studies charged the patient directly for the POCT. Patients were most commonly recruited into the studies via clinician referral or through pharmacy list searches, with only one of the seven observational studies recruiting patients opportunistically.

Quality assessment

The overall methodological quality (online Figures 1-3) was moderate across the five prospective controlled trials, with three studies exhibiting a high risk of detection bias (lack of blinding of the outcome assessors) and an unclear risk of reporting bias (no study protocol available).(22,24,27) The non-randomised and before-after studies generally did not provide sample size justifications, and four of these studies did not account for confounding variables in patient selection.(15,17,19,20) For the single case-control study, the comparability of cases and controls was scored as "high risk", due to significant differences in the selection procedure.(26)

For peer review only

For peer review only - http://bmjopen.bmj.com/site/about/guidelines.xhtml

Outcomes and Tests used

Malaria

Three randomized controlled trials (n= 20,699) investigated the use of POCT in the context of Malaria.(23–25) All three studies reported the difference in total use of anti-malarial drugs between POCT and usual care groups (Figure 2). Utilisation of POCT in a pharmacy setting reduced total anti-malarial use (risk ratio (RR) 0.58, 95% CI 0.54 to 0.62) over usual care, however pooled estimates exhibited significant statistical heterogeneity (I^2= 90%). For context, usual care most commonly consisted of pharmacists making decisions to supply anti-malarial drugs using their clinical judgement or other parameters such as the patient's temperature, without a rapid diagnostic test.

Two studies reported the difference between appropriately dispensed anti-malarial drugs (defined as: anti-malaria indicated, anti-malarial given) given to patients receiving POCT or usual care.(23,25) These trials found that the risk of receiving inappropriate antimalarial treatment was reduced in the pharmacy POCT group compared to usual care (RR 0.34 95% CI 0.31-0.37, I^2= 76%).

International Normalised ratio (INR)

Five studies (n= 1,018) investigated the use of POCT in the context of INR testing – four pre/post observational studies,(15–17,27) and one retrospective cohort study (Figure 3).(26) Pooled analysis of the pre/post observational studies showed no clear benefit for POCT in pharmacies for INR control, as measured by percentage of time in therapeutic range (TTR) of target INR.(28) Mean difference in percentage TTR between POCT and usual care group was 7.99% (95%CI 0.74% to 16.71%; I^2 = 99%) in favour of pharmacist POCT. The single retrospective cohort study found an increase of 19.90% (95% CI 12.45 to 27.35%) in favour of pharmacist POCT.

Lipids.

Three studies investigated the use of POCT in pharmacies with regards to lipid monitoring. Two studies were pre-post observational studies,(20,21) and one was a randomised trial (Supplementary Figure 1).(22)

For peer review only - http://bmjopen.bmj.com/site/about/guidelines.xhtml

Total cholesterol (TC) was investigated in all three studies. The RCT showed no significant difference in TC levels over usual care at six months (mean difference -7.80, 95% CI -19.65 to 4.05mg/dL;) whereas the pooled analysis of the two observational studies did suggest a significant decrease in TC between baseline and two year follow-up (mean difference -29.63mg/dL, 95% CI -35.29 to -23.98mg/dL; $I^2 = 0\%$).

At two years, meta-analysis showed a significant decrease in LDL (low-density lipoprotein) cholesterol between POC and usual care groups (mean difference -28.90mg/dL, 95% CI -40.74 to -9.65mg/dL; $I^2 = 70\%$). Furthermore, an increase in HDL (high-density lipoprotein) cholesterol was observed, however this was non-significant (mean difference 3.96mg/dL, 95% CI -0.80 to +8.72mg/dL; $I^2 = 77\%$).

Mean TG (triglycerides), LDL and HDL cholesterol were measured in the two observational studies.(20,21) Mean TG concentration was reduced from baseline levels after two-year follow-up (mean difference -21.68, 95% CI -34.74 to -8.61mg/dL; $I^2 = 0\%$).

<u>HbA1c</u>

Two observational studies (n=226) investigated the effect of POCT on HbA1c control among diabetic patients.(18,19) The studies did not find a significant difference between baseline and follow-up HbA1c measurements (-1.02%, 95% CI -2.59% to 0.54%; $I^2 = 96\%$, Supplementary Figure 2).

For peer review only - http://bmjopen.bmj.com/site/about/guidelines.xhtml

DISCUSSION

Summary of findings

We identified 13 studies including over 23,000 patients evaluating the clinical impact of POCT based in pharmacies. The available evidence was generally of poor methodological quality, and only 4/13 studies were randomised controlled trials.

The findings of this review suggest that pharmacy-based POCT may be useful in guiding appropriate anti-malaria prescribing, particularly in low resource settings. Further use of POCT, such as in lipid control, appeared to show some promise although the limited number of studies meant this could not be confirmed and the practical application of these tests in practice were unclear. There was no evidence that the delivery of POCT alone improved INR time-in-therapeutic-range or HbA1c levels in the community pharmacy setting.

Strengths and limitations

This review provides a timely and comprehensive overview regarding the current evidence related to POCT in pharmacies. The search and review strategy meant that we were unlikely to have missed large numbers of eligible studies. However, studies where the use of POCTs were embedded among other interventions were not included in this review given the difficulty in isolating the effect of the POCT on the outcomes measured. Such studies may also provide useful information regarding the contribution of POCT to clinical outcomes.

The majority of included studies were observational and were generally of poor methodological quality (Online Figures 1-3). Although this limits our understanding of the clinical benefits (or harms) of these POCTs delivered in pharmacies, it highlights a need for high quality primary studies in this area of clinical practice. Furthermore, the primary literature included in this review were of limited clinical scope, covering only four therapeutic areas (anti-malarial drugs, Hba1c, INR and lipid levels). There was no data on areas such as acute infections that commonly present to community pharmacies - something that NHS commissioners have considered introducing into community pharmacies in the UK.(7) There is therefore no strong evidence for the use of POCT for either chronic disease monitoring or acute disease diagnosis in the community pharmacy setting at present. In addition, none of the included studies were conducted in the UK, making the generalisability to UK primary care challenging.

For peer review only - http://bmjopen.bmj.com/site/about/guidelines.xhtml
For peer review only

Comparison with previous literature

A systematic review published in 2018 by Buss et al. aimed to summarise the literature related to both analytical quality and effectiveness of POCT in pharmacies.(10) Unlike our review, Buss et al included studies where pharmacy POCT performance was compared to corresponding laboratory results. Our review included the two studies contained within Buss et al. that compared pharmacy POCT with a control.(15,17) In addition to these, we were able to include a further ten papers.

In 2016, the largest pharmacy chain in the UK conducted a single arm feasibility study in 35 pharmacies - offering POCT for group A streptococcal pharyngitis.(8) After CENTOR scoring, patients testing positive for group A streptococci were offered antibiotic treatment at the pharmacy. A total of 149/367 (40.6%) patients received a throat swab, and of these, 36/149 (24.2%) were positive for group A streptococci. Antibiotics were supplied to 9.8% (*n*=36/367) of patients accessing the service. The study concluded that it was feasible to deliver such a service. The study did not report any clinical outcomes and therefore was not included in this review, although the number of GP consultations prevented and the reduction in antibiotic use was estimated based on patient self-reporting. Our systematic review has demonstrated that, to date, the impact on both clinical outcomes and total healthcare utilisation are yet to be established with regards to acute bacterial infections from a pharmacy setting.

Implications for clinical practice

Policy makers have identified community pharmacies as appropriate locations for extended healthcare delivery.(30) This is due in part to the strain on other areas of the health system, the convenience offered to patients who can see a pharmacist without an appointment, and the fact that pharmacists are highly trained in the safe use of medications. However, this systematic review has highlighted that extending the role of pharmacists to delivering POCT may require further assessment before large-scale rollout. Furthermore, to provide POCT in the future, pharmacists and their staff will require specific training on the tests they provide and in managing the results appropriately. Other considerations such as the practicality and safety associated with handling bodily fluids in pharmacies will also require resolution (e.g. a suitable location for patients to provide a urine sample), in addition to having an appropriate medico-

For peer review only

For peer review only - http://bmjopen.bmj.com/site/about/guidelines.xhtml

legal framework to allow pharmacists to deliver such interventions. Additional considerations include the source of funding for such services (local, national or patient funded). The Strep A feasibility study mentioned above was paid for by patients.(8) How such a model would fit in with the current NHS is another consideration that may require extensive stakeholder deliberation, cost-effectiveness analyses and health inequalities assessment.

Furthermore, the application of the findings from the lipid control studies in this review may be limited, given that in the UK, lipids are most commonly managed on the basis of overall cardiovascular disease (CVD) risk scoring,(31) rather than a stand-alone clinic. Therefore, there is likely to be limited application of POCT for lipids alone, unless it is conducted as part of a CVD risk assessment in the pharmacy.

A policy document presented to the American Pharmacist Association policy committee in 2015-16 outlined the following as potential barriers to the uptake of POCT by pharmacists:(32)

1. Lack of payment mechanisms.
2. Lack of standardised training/education across the profession.
3. Lack of standardised documentation systems and follow-up procedures.
4. Inconsistency in providing POCT services (post-code lottery).
5. Perceived pushback from medical and other related health professionals.

In addition to the operational and practical barriers stated above, this review has highlighted that lack of evidence of effectiveness and healthcare utilisation may also be contributing factors to the lack of commissioning and uptake.

Conclusion and recommendations

The few studies available suggest some promise in the use of pharmacy-based POCT for appropriate anti-malarial dispensing in low-resource settings, and for the control of blood lipids – however even these results require cautious interpretation given the heterogeneity observed and lack of evidence on clinically relevant outcomes. This systematic review has identified gaps in the literature regarding the evidence for use of POCT in pharmacies, particularly in areas such as the triage and treatment of common acute bacterial or viral respiratory tract infections, where no evidence was found.

For peer review only - http://bmjopen.bmj.com/site/about/guidelines.xhtml

Future studies could consider non-inferiority of clinical outcomes versus usual care if the intervention is shown to be safe and cost-effective. Other outcomes such as patient access to care and re-presentation to general practice/out of hours care should also be carefully recorded in future studies.

Policy recommendations for the introduction of POCT in pharmacies should be informed by well-conducted randomised controlled trials and economic analyses of the specific condition(s) being considered. Until such time as these data become available, caution is required before the widespread roll-out of POCT.

For peer review only

For peer review only - http://bmjopen.bmj.com/site/about/guidelines.xhtml

Contribution statement

JYV and AA conceived the study. AA, JYV did data extraction. AA and JYV performed the analyses, which were discussed with AVdB, GH, RM, JS. JYV and AA drafted this report and AVdB, GH, RM, JS co-drafted and commented on the final version. All authors had full access to all of the data (including statistical reports and tables) in the study and take responsibility for the integrity of the data and the accuracy of the data analysis. JYV affirms that the manuscript is an honest, accurate, and transparent account of the study being reported; that no important aspects of the study have been omitted and will act as guarantor. All authors have read and approved the final manuscript.

Competing interests

All authors have completed the ICMJE uniform disclosure form at www.icmje.org/coi_disclosure.pdf and declare: no support from any organisation for the submitted work; no financial relationships with any organisations that might have an interest in the submitted work in the previous three years; no other relationships or activities that could appear to have influenced the submitted work.

Funding

JYV, GH, AVdB are supported through the National Institute for Health Research (NIHR) Community Healthcare MedTech and IVD Co-operative. AA receives funding from the NIHR SPCR. RJM and JS receive support from Oxford NIHR CLAHRC. JS also receives funding from the Wellcome Trust/Royal Society via a Sir Henry Dale Fellowship (ref: 211182/Z/18/Z) and the NIHR School for Primary Care Research. RJM is a NIHR Senior Investigator.

Data sharing statement

All data for these analyses are included in the manuscript or online appendices. No additional data are available.

ACKNOWLEDGEMENTS

The views expressed are those of the authors and not necessarily those of the National Health Service (NHS), the National Institute for Health Research (NIHR), or the Department of Health and Social Care. JYV had full access to all the data in the study and takes responsibility for the integrity of the data and the accuracy of the data analysis.

For peer review only

For peer review only - http://bmjopen.bmj.com/site/about/guidelines.xhtml

FIGURES & SUPPLEMENTARY FILES

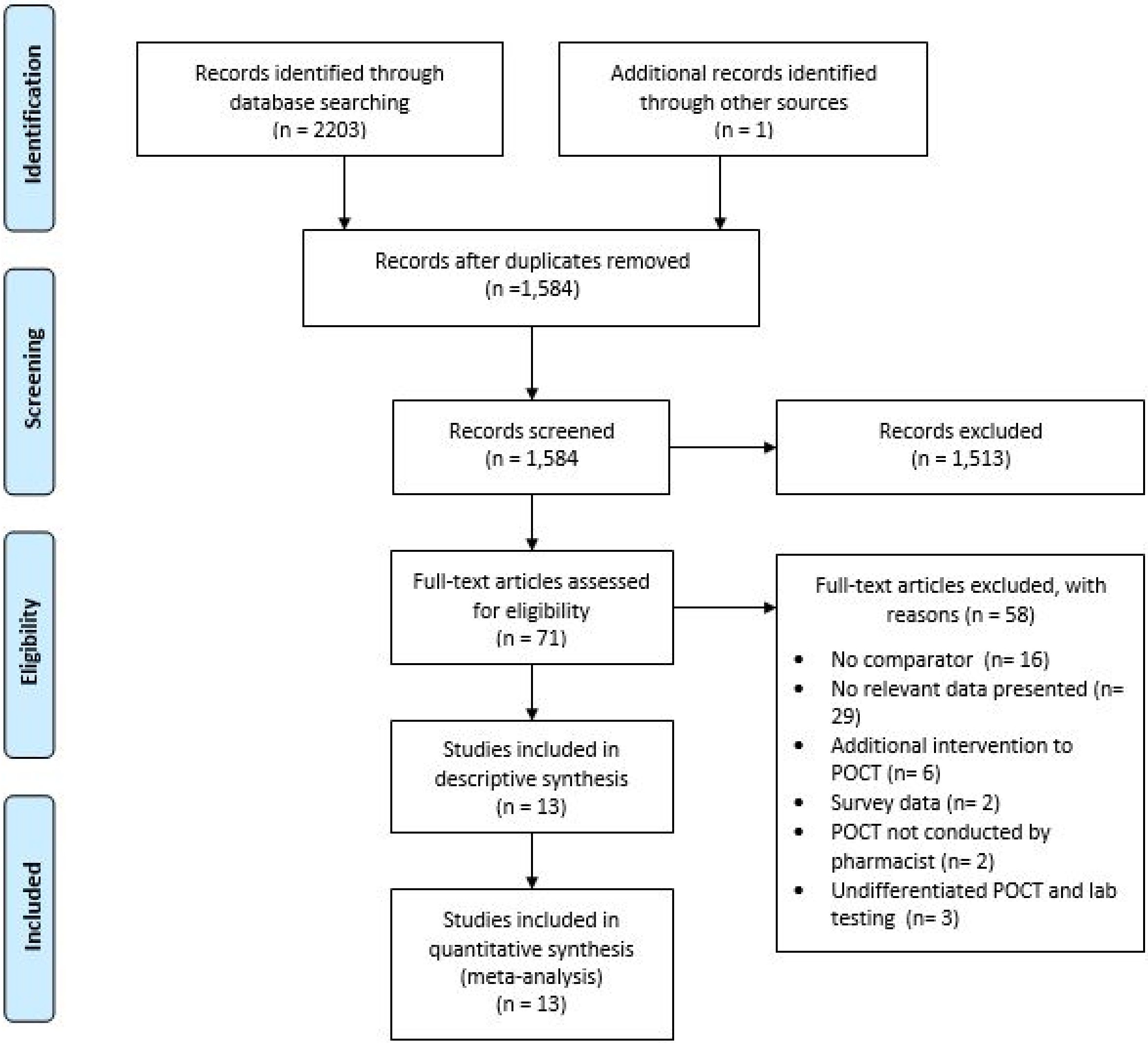

Figure 1 - PRISMA flow diagram

For peer review only - http://bmjopen.bmj.com/site/about/guidelines.xhtml

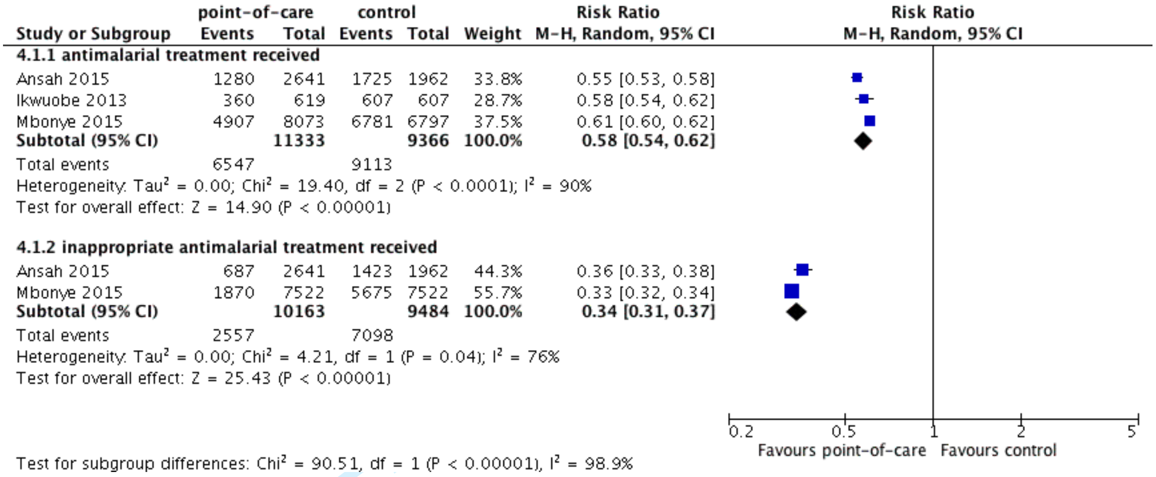

Figure 2 – Anti-malarials

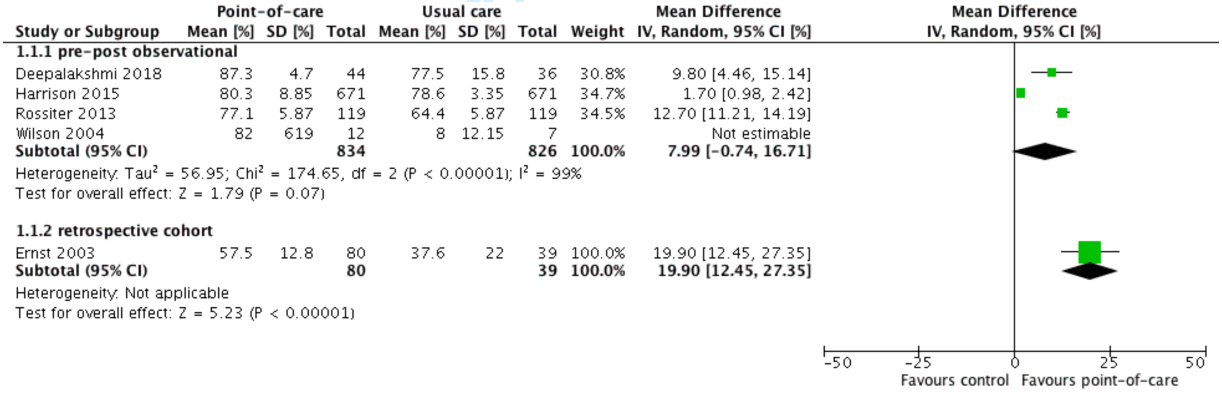

Figure 3 – INR time in therapeutic range (TTR)

For peer review only

For peer review only - http://bmjopen.bmj.com/site/about/guidelines.xhtml

Study author & year	Study design	Country	POC test (condition or level monitored)	Test conducted by	Location of POCT	Total population (n)	Mean (SD) or median age (IQR) (I/C), years	Gender (% M)
Ansah 2015	Cluster RCT	Ghana	Malaria testing (CareStart Malaria HRP2 (Pf), Apacor)	Chemical seller	Private drug retail shops/chemical shops	4,603	15 (6-29)/ 19 (6-32)	51%
Mbonye 2015	Cluster RCT	Uganda	Malaria testing (First Response Malaria Ag. Combo Rapid Diagnostic Test, Premier Med Corp)	Drug shop vender	"Drug shops"	15,517	NA	48.3%
Ikwuobe 2013	RCT	Nigeria	Malaria testing (SD Bioline Malaria Antigen Pf, Alere)	Pharmacist	Community pharmacy	1,226	30.8 (NA)	48.3%
Al Hamarneh 2013	Pre-post observational	Canada	Hba1c (DCA Vantage, Siemens)	Independent prescribing pharmacist	Community pharmacies	100	64 (10.4)	58%
Oyetayo 2011	Pre-post observational	USA	Hba1c (device not specified)	Pharmacist	Community pharmacy	126	NA	NA
Gerrald 2010	Pre-post observational	USA	Lipid profile testing (Cholestech LDX Analyzer, Alere)	Pharmacist	Outpatient clinic	81	64.9 (6.9)	79.1%
Peterson 2004	RCT	Australia	Total cholesterol (Accutrend GC, Roche Diagnostics)	Pharmacist	Pharmacist visiting at home	81	63.5 (12.1)/ 65.5 (11.0)	63%
Bluml 2000	Pre-post observational	USA	Lipid profile testing (Cholestech LDX Analyzer, Alere)	Pharmacist	Community Pharmacy	397	57 (NA)	48%
Deepalakshmi 2018	Prospective controlled parallel trial	India	INR (CoaguChek XS Plus, Roche Diagnostics)	Pharmacist	Community pharmacy	80	61.4 (3.1)	74.4%
Harrison 2015	Pre-post observational	New Zealand	INR (CoaguChek XS Plus, Roche Diagnostics)	Pharmacist	Community pharmacy	671	72 (13-97)	62.4%
Rossiter 2013	Pre-post observational	Canada	INR (CoaguCheck XS Machine, Roche Diagnostics)	Pharmacist	Pharmacist-led POC clinic	119	78.8 (NA)	48.7%
Wilson 2004	Pre-post observational	USA	INR (Coaguchek-S, Roche Diagnostics)	Pharmacist	Community pharmacy	19	61 (NA)	68%
Ernst 2003	Retrospective cohort	USA	INR (CoaguCheck (Boehringer Mannheim)	Pharmacist	Pharmacist led outpatient clinic	129	76.6 (12.8)	45%

Table 1: Baseline characteristics POC: point-of-care, SD: standard deviation, IQR: interquartile range, I/C: intervention group/control group, M: Male, INR: International Normalised Ratio, NA: Not Available, RCT: randomised controlled trial. Studies grouped according to point of care test used and chronologically within each test (most recent first).

For peer review only

Supplementary File 1: Medline search strategy

# ▲	Searches
1	((rapid$ or same time or same visit or near patient or portable or handheld or hand-held) adj3 (test$ or analys$ or analyz$ or measure$ or assay$ or monitor* or device*)).ti,ab.
2	(fingerprick or finger prick).tw.
3	(poc or poct or "point of care").tw.
4	point-of-care systems/ or point-of-care testing/
5	1 or 2 or 3 or 4
6	Community Pharmacy Services/
7	Pharmacists/
8	(pharmacy or pharmacies or pharmacist?).ti,ab.
9	6 or 7 or 8
10	5 and 9
11	randomized controlled trial.pt.
12	controlled clinical trial.pt.
13	randomized.ab.
14	placebo.ab.
15	drug therapy.fs.
16	randomly.ab.
17	trial.ab.
18	groups.ab.
19	11 or 12 or 13 or 14 or 15 or 16 or 17 or 18
20	(animals not (humans and animals)).sh.
21	19 not 20
22	10 and 21
23	10 not 22

For peer review only

Online Figure 1 - Risk of bias for pre-post studies

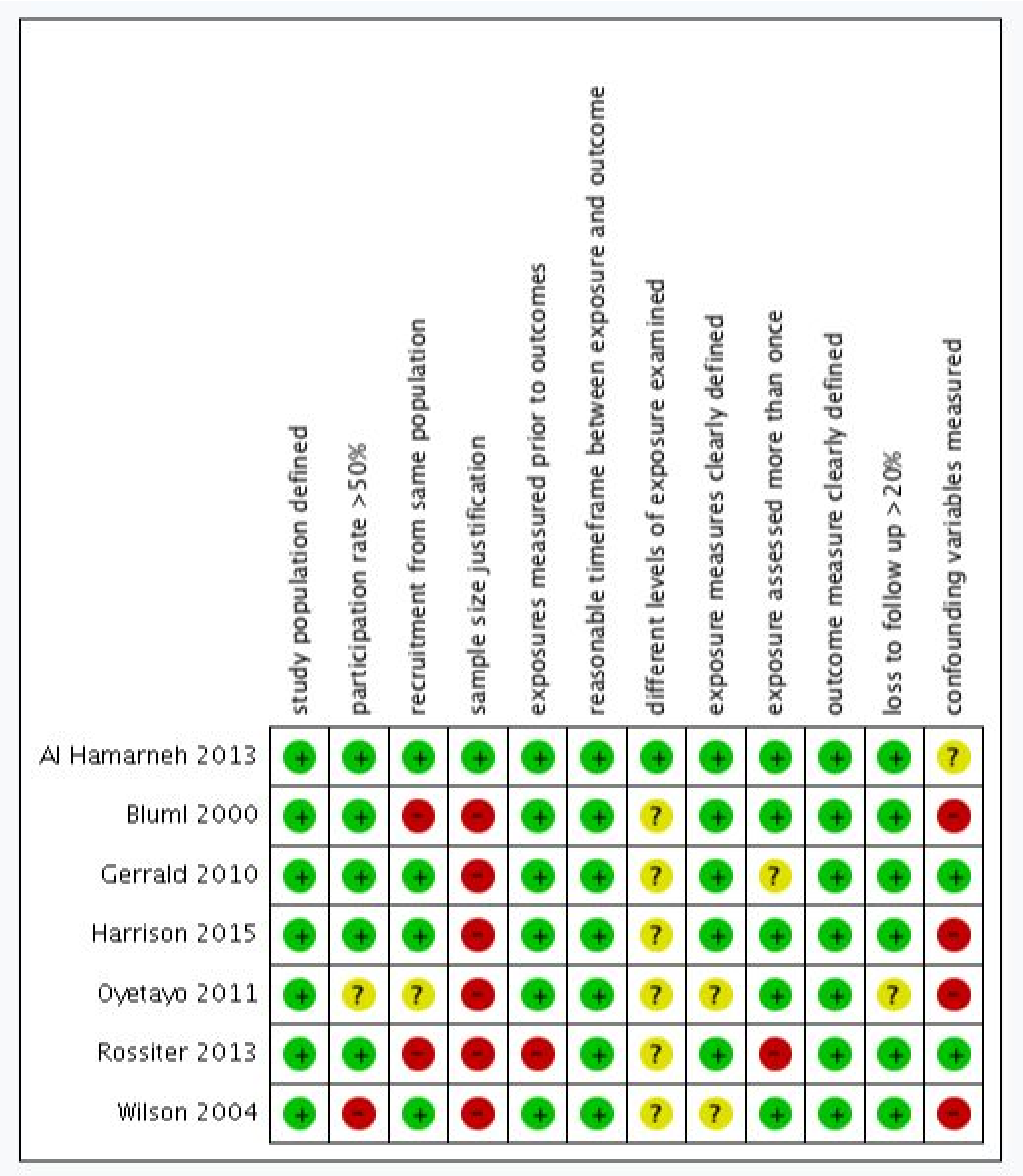

Online Figure 2 - Risk of bias for retrospective case/control study

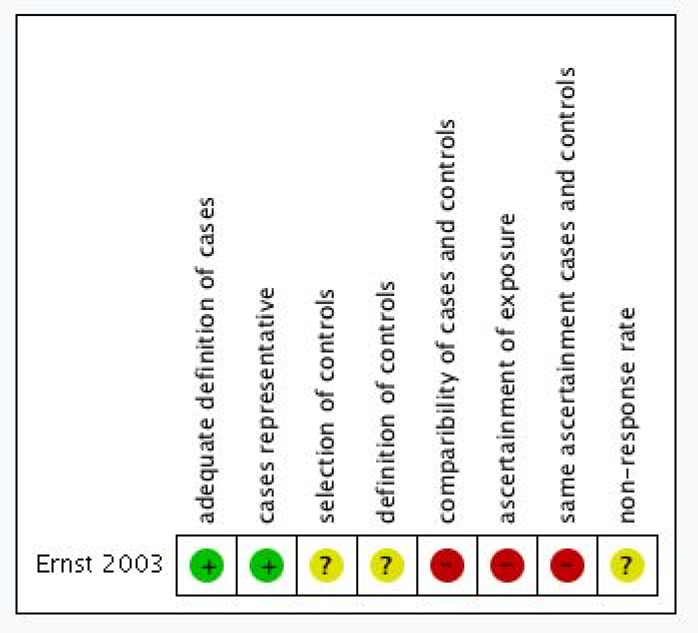

Online Figure 3 - Risk of bias for prospective controlled trials

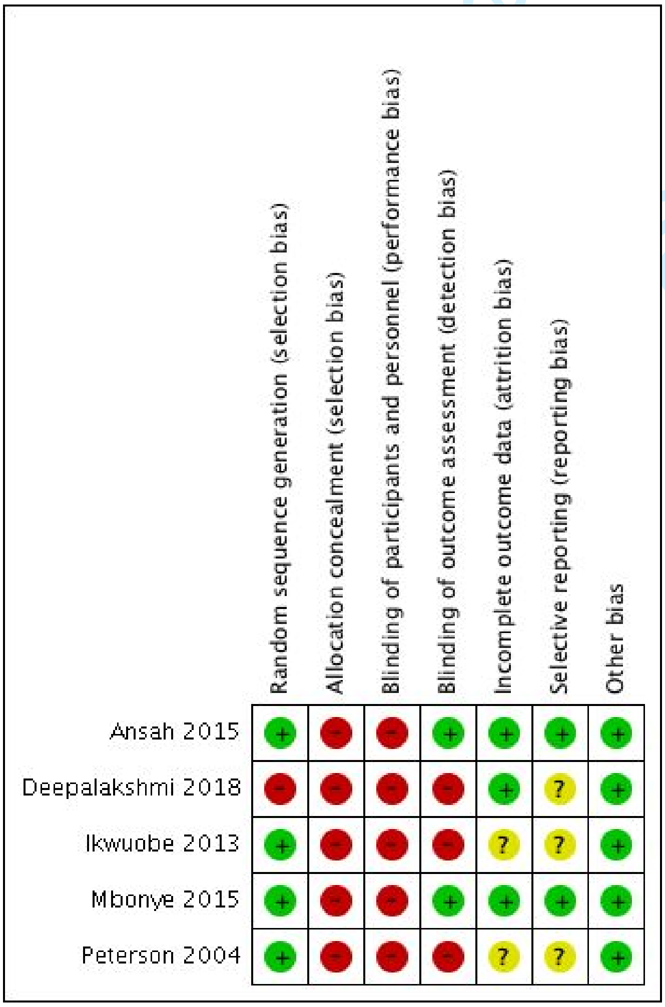

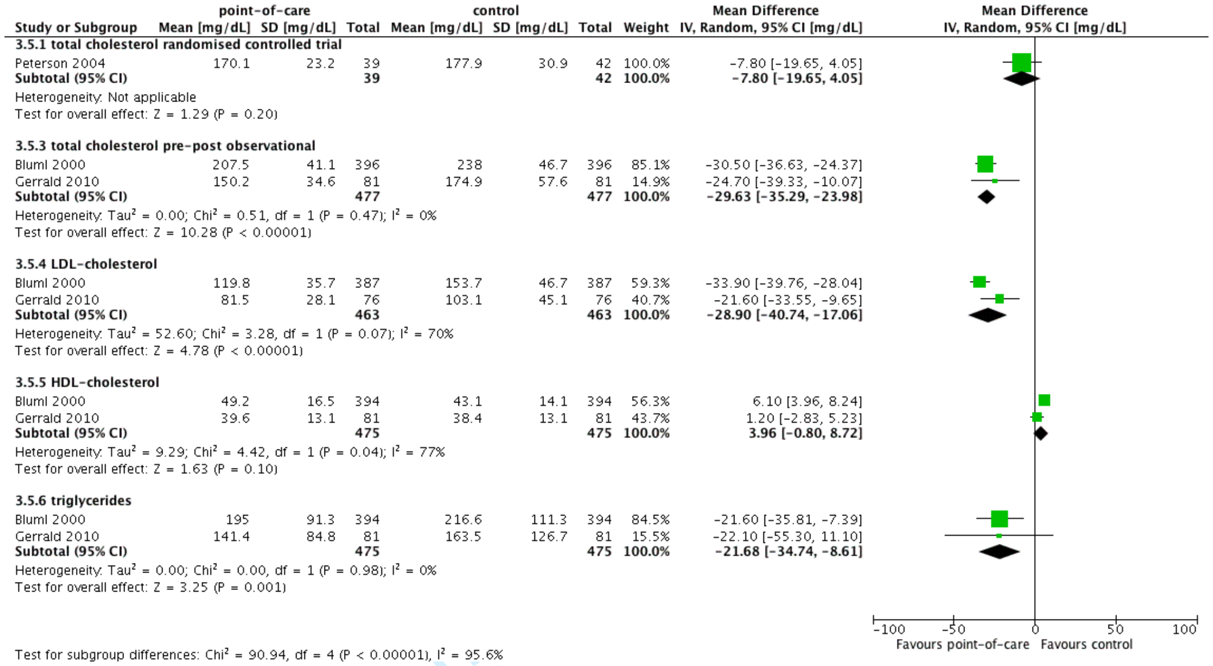

Online figure 4 – Lipids (Total cholesterol, HDL cholesterol, LDL cholesterol and triglycerides)

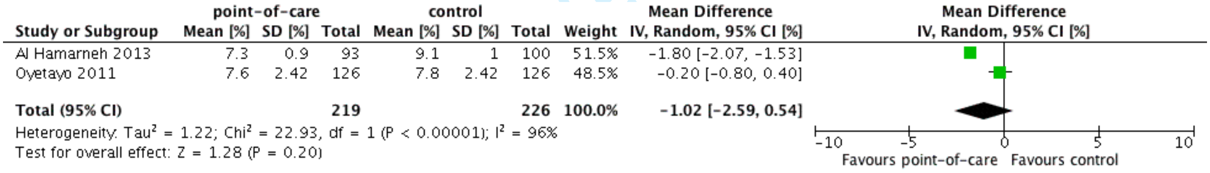

Online figure 5 - HbA1c

References:

1. Schols AMR, Dinant G-J, Hopstaken R, Price CP, Kusters R, Cals JWL. International definition of a point-of-care test in family practice: a modified e-Delphi procedure. Fam Pract [Internet]. 2018 Jul 23;35(4):475–80. Available from: http://www.ncbi.nlm.nih.gov/pubmed/29385437
2. National Institute of Biomedical Imaging and Bioengineering/National Heart, Lung, and Blood Institute/National Science Foundation Workshop Faculty CP, Price CP, Kricka LJ. Improving healthcare accessibility through point-of-care technologies. Clin Chem [Internet]. 2007 Sep 1;53(9):1665–75. Available from: http://www.clinchem.org/cgi/doi/10.1373/clinchem.2006.084707
3. Abel G. Current status and future prospects of point-of-care testing around the globe. Expert Rev Mol Diagn [Internet]. 2015 Jul 3;15(7):853–5. Available from: http://www.ncbi.nlm.nih.gov/pubmed/26109315
4. Pharmaceutical Services Negotiating Committee, Pharmacy Voice. Community Pharmacy Forward View. 2016; Available from: http://psnc.org.uk/wp-content/uploads/2016/08/CPFV-Aug-2016.pdf
5. Hobbs FDR, Bankhead C, Mukhtar T, Stevens S, Perera-Salazar R, Holt T, et al. Clinical workload in UK primary care: a retrospective analysis of 100 million consultations in England, 2007–14. Lancet [Internet]. 2016;387(10035):2323–30. Available from: http://www.sciencedirect.com/science/article/pii/S0140673616006206
6. Howick J, Cals JWL, Jones C, Price CP, Plüddemann A, Heneghan C, et al. Current and future use of point-of-care tests in primary care: an international survey in Australia, Belgium, The Netherlands, the UK and the USA. BMJ Open [Internet]. 2014;4. Available from: http://bmjopen.bmj.com/
7. Lewis G. NHS England to roll out Boots sore throat scheme. Chemist and Druggist [Internet]. 2016; Available from: https://www.chemistanddruggist.co.uk/news/nhs-england-roll-out-boots's-sore-throat-scheme
8. Thornley T, Marshall G, Howard P, Wilson APR. A feasibility service evaluation of screening and treatment of group A streptococcal pharyngitis in community pharmacies. J Antimicrob Chemother [Internet]. 2016 Nov 1;71(11):3293–9. Available from: https://academic.oup.com/jac/article-lookup/doi/10.1093/jac/dkw264
9. McKenna C, Burch J, Suekarran S, Walker S, Bakhai A, Witte K, et al. A systematic review and economic evaluation of the clinical effectiveness and cost-effectiveness of aldosterone antagonists for postmyocardial infarction heart failure. Health Technol Assess (Rockv) [Internet]. 2010 May;14(24). Available from: https://www.journalslibrary.nihr.ac.uk/hta/hta14240/
10. Buss VH, Deeks LS, Shield A, Kosari S, Naunton M. Analytical quality and effectiveness of point-of-care testing in community pharmacies: A systematic literature review. Res Soc Adm Pharm [Internet]. 2018 Jul 20; Available from: https://www.sciencedirect.com/science/article/pii/S1551741118303152
11. Higgins J, Green S. Cochrane Handbook for systematic reviews of interventions version. In: The Cochrane Collaboration [Internet]. 5.1.0. 2011. p. 2–3. Available from: www.handbook.cochrane.org.
12. GA Wells, B Shea, D O'Connell, J Peterson, V Welch, M Losos PT. The Newcastle-Ottawa Scale (NOS) for assessing the quality of non-randomized studies in meta-analyses [Internet]. Ottawa Hospital Research Institute. 2013. Available from: http://www.ohri.ca/programs/clinical_epidemiology/oxford.asp
13. Higgins JP, Thompson SG, Spiegelhalter DJ. A re-evaluation of random-effects meta-analysis. J R Stat Soc Ser A Stat Soc [Internet]. 2009;172(1):137–59. Available from:

For peer review only

http://www.ncbi.nlm.nih.gov/pubmed/19381330
14. Egger M, Davey Smith G, Schneider M, Minder C. Bias in meta-analysis detected by a simple, graphical test. BMJ [Internet]. 1997 Sep 13 [cited 2017 Dec 11];315(7109):629–34. Available from: http://www.ncbi.nlm.nih.gov/pubmed/9310563
15. Harrison J, Shaw JP, Harrison JE. Anticoagulation management by community pharmacists in New Zealand: an evaluation of a collaborative model in primary care. Int J Pharm Pract [Internet]. 2015 Jun 1;23(3):173–81. Available from: http://doi.wiley.com/10.1111/ijpp.12148
16. Rossiter J, Soor G, Telner D, Aliarzadeh B, Lake J. A Pharmacist-Led Point-of-Care INR Clinic: Optimizing Care in a Family Health Team Setting. Int J Family Med [Internet]. 2013;2013:691454. Available from: http://www.ncbi.nlm.nih.gov/pubmed/24455250
17. Wilson SJ-A, MacLellan E, Cox JL, Meek W, Monette K, Morash T, et al. A Pilot Study Evaluating the Feasibility of Monitoring Oral Anticoagulant Therapy with Point-of-Care Testing in a Community Pharmacy. Can J Hosp Pharm [Internet]. 2004;57(3). Available from: https://www.cjhp-online.ca/index.php/cjhp/article/view/369
18. Al Hamarneh YN, Charrois T, Lewanczuk R, Tsuyuki RT. Pharmacist intervention for glycaemic control in the community (the RxING study). BMJ Open [Internet]. 2013 Sep 24;3(9):e003154. Available from: http://www.ncbi.nlm.nih.gov/pubmed/24068762
19. Oyetayo OO, James C, Martinez A, Roberson K, Talbert RL. The Hispanic Diabetes Management Program: Impact of community pharmacists on clinical outcomes. J Am Pharm Assoc [Internet]. 2011 Sep;51(5):623–6. Available from: http://www.ncbi.nlm.nih.gov/pubmed/21896461
20. Bluml BM, McKenney JM, Cziraky MJ. Pharmaceutical care services and results in project ImPACT: hyperlipidemia. J Am Pharm Assoc (Wash) [Internet]. 40(2):157–65. Available from: http://www.ncbi.nlm.nih.gov/pubmed/10730019
21. Gerrald KR, Dixon DL, Barnette DJ, Williams VG. Evaluation of a pharmacist-managed lipid clinic that uses point-of-care lipid testing. J Clin Lipidol [Internet]. 2010 Mar;4(2):120–5. Available from: http://www.ncbi.nlm.nih.gov/pubmed/21122639
22. Peterson GM, Fitzmaurice KD, Naunton M, Vial JH, Stewart K, Krum H. Impact of pharmacist-conducted home visits on the outcomes of lipid-lowering drug therapy. J Clin Pharm Ther [Internet]. 2004 Feb;29(1):23–30. Available from: http://www.ncbi.nlm.nih.gov/pubmed/14748894
23. Ansah EK, Narh-Bana S, Affran-Bonful H, Bart-Plange C, Cundill B, Gyapong M, et al. The impact of providing rapid diagnostic malaria tests on fever management in the private retail sector in Ghana: a cluster randomized trial. BMJ [Internet]. 2015 Mar 4;350:h1019. Available from: http://www.ncbi.nlm.nih.gov/pubmed/25739769
24. Ikwuobe JO, Faragher BE, Alawode G, Lalloo DG. The impact of rapid malaria diagnostic tests upon anti-malarial sales in community pharmacies in Gwagwalada, Nigeria. Malar J [Internet]. 2013 Dec 30;12(1):380. Available from: https://malariajournal.biomedcentral.com/articles/10.1186/1475-2875-12-380
25. Mbonye AK, Magnussen P, Lal S, Hansen KS, Cundill B, Chandler C, et al. A Cluster Randomised Trial Introducing Rapid Diagnostic Tests into Registered Drug Shops in Uganda: Impact on Appropriate Treatment of Malaria. Gosling RD, editor. PLoS One [Internet]. 2015 Jul 22;10(7):e0129545. Available from: http://www.ncbi.nlm.nih.gov/pubmed/26200467
26. Ernst ME, Brandt KB. Evaluation of 4 Years of Clinical Pharmacist Anticoagulation Case Management in a Rural, Private Physician Office. J Am Pharm Assoc [Internet]. 2003 Sep 1;43(5):630–6. Available from:

https://www.sciencedirect.com/science/article/pii/S1544319115319270
27. Deepalakshmi M D, Joseph C, Jacob C, Arun K P A, Ponnusankar S P. Management of Warfarin Therapy: A New Point-Of-Care Monitoring Service by Community Pharmacists in India. J Young Pharm [Internet]. 2018 [cited 2019 Jun 21];10(3):350–3. Available from: www.jyoungpharm.org%7Cwww.phcog.net
28. Schmitt L, Speckman J, Ansell J. Quality Assessment of Anticoagulation Dose Management: Comparative Evaluation of Measures of Time-in-Therapeutic Range [Internet]. Vol. 15, Journal of Thrombosis and Thrombolysis. Kluwer Academic Publishers; 2003. Available from: https://link.springer.com/content/pdf/10.1023%2FB%3ATHRO.0000011377.78585.63.pdf
29. Schols AMR, Stakenborg JPG, Dinant G-J, Willemsen RTA, Cals JWL. Point-of-care testing in primary care patients with acute cardiopulmonary symptoms: a systematic review. Fam Pract [Internet]. 2018 Jan 16;35(1):4–12. Available from: http://www.ncbi.nlm.nih.gov/pubmed/28985344
30. National Health Service. Five Year Forward View [Internet]. 2014. Available from: https://www.england.nhs.uk/wp-content/uploads/2014/10/5yfv-web.pdf
31. NICE. Cardiovascular disease : risk assessment and reduction , including lipid modification. NICE Guidel [Internet]. 2016;1(July 2014):1–44. Available from: https://www.nice.org.uk/guidance/cg181/chapter/Appendix-A-Grouping-of-statins
32. Marsha Gilbreath. Point-of-Care Testing Background Paper Prepared for the 2015–2016 APhA Policy Committee. 2013.

For peer review only

PRISMA 2009 Checklist

Section/topic	#	Checklist item	Reported on page #
**TITLE**			
Title	1	Identify the report as a systematic review, meta-analysis, or both.	1
**ABSTRACT**			
Structured summary	2	Provide a structured summary including, as applicable: background; objectives; data sources; study eligibility criteria, participants, and interventions; study appraisal and synthesis methods; results; limitations; conclusions and implications of key findings; systematic review registration number.	2
**INTRODUCTION**			
Rationale	3	Describe the rationale for the review in the context of what is already known.	4
Objectives	4	Provide an explicit statement of questions being addressed with reference to participants, interventions, comparisons, outcomes, and study design (PICOS).	4,6
**METHODS**			
Protocol and registration	5	Indicate if a review protocol exists, if and where it can be accessed (e.g., Web address), and, if available, provide registration information including registration number.	7
Eligibility criteria	6	Specify study characteristics (e.g., PICOS, length of follow-up) and report characteristics (e.g., years considered, language, publication status) used as criteria for eligibility, giving rationale.	5
Information sources	7	Describe all information sources (e.g., databases with dates of coverage, contact with study authors to identify additional studies) in the search and date last searched.	5
Search	8	Present full electronic search strategy for at least one database, including any limits used, such that it could be repeated.	5
Study selection	9	State the process for selecting studies (i.e., screening, eligibility, included in systematic review, and, if applicable, included in the meta-analysis).	5
Data collection process	10	Describe method of data extraction from reports (e.g., piloted forms, independently, in duplicate) and any processes for obtaining and confirming data from investigators.	6
Data items	11	List and define all variables for which data were sought (e.g., PICOS, funding sources) and any assumptions and simplifications made.	6
Risk of bias in individual studies	12	Describe methods used for assessing risk of bias of individual studies (including specification of whether this was done at the study or outcome level), and how this information is to be used in any data synthesis.	6
Summary measures	13	State the principal summary measures (e.g., risk ratio, difference in means).	6
Synthesis of results	14	Describe the methods of handling data and combining results of studies, if done, including measures of consistency (e.g., I^2) for each meta-analysis.	6, 7

For peer review only

For peer review only - http://bmjopen.bmj.com/site/about/guidelines.xhtml

PRISMA 2009 Checklist

Section/topic	#	Checklist item	Reported on page #
Risk of bias across studies	15	Specify any assessment of risk of bias that may affect the cumulative evidence (e.g., publication bias, selective reporting within studies).	6
Additional analyses	16	Describe methods of additional analyses (e.g., sensitivity or subgroup analyses, meta-regression), if done, indicating which were pre-specified.	6
**RESULTS**			
Study selection	17	Give numbers of studies screened, assessed for eligibility, and included in the review, with reasons for exclusions at each stage, ideally with a flow diagram.	8
Study characteristics	18	For each study, present characteristics for which data were extracted (e.g., study size, PICOS, follow-up period) and provide the citations.	8
Risk of bias within studies	19	Present data on risk of bias of each study and, if available, any outcome level assessment (see item 12).	8
Results of individual studies	20	For all outcomes considered (benefits or harms), present, for each study: (a) simple summary data for each intervention group (b) effect estimates and confidence intervals, ideally with a forest plot.	9
Synthesis of results	21	Present results of each meta-analysis done, including confidence intervals and measures of consistency.	9,10 table 1, figures
Risk of bias across studies	22	Present results of any assessment of risk of bias across studies (see Item 15).	Suppl fig 1
Additional analysis	23	Give results of additional analyses, if done (e.g., sensitivity or subgroup analyses, meta-regression [see Item 16]).	Supple fig 2-5
**DISCUSSION**			
Summary of evidence	24	Summarize the main findings including the strength of evidence for each main outcome; consider their relevance to key groups (e.g., healthcare providers, users, and policy makers).	11
Limitations	25	Discuss limitations at study and outcome level (e.g., risk of bias), and at review-level (e.g., incomplete retrieval of identified research, reporting bias).	11
Conclusions	26	Provide a general interpretation of the results in the context of other evidence, and implications for future research.	13,14
**FUNDING**			
Funding	27	Describe sources of funding for the systematic review and other support (e.g., supply of data); role of funders for the systematic review.	15

For peer review only

PRISMA 2009 Checklist

From: Moher D, Liberati A, Tetzlaff J, Altman DG, The PRISMA Group (2009). Preferred Reporting Items for Systematic Reviews and Meta-Analyses: The PRISMA Statement. PLoS Med 6(6): e1000097. doi:10.1371/journal.pmed1000097

For more information, visit: **www.prisma-statement.org**.

For peer review only

BMJ Open

Impact of point-of-care tests in community pharmacies: a systematic review and meta-analysis

Journal:	*BMJ Open*
Manuscript ID	bmjopen-2019-034298.R1
Article Type:	Original research
Date Submitted by the Author:	27-Feb-2020
Complete List of Authors:	Albasri, Ali; University of Oxford , Nuffield Department of Primary Care Health Sciences<br>Van Den Bruel, Ann; KU Leuven Department of Public Health and Primary Care<br>Hayward, Gail; University of Oxford, Nuffield Department of Primary Care Health Sciences<br>McManus, Richard; University of Oxford, Dept of Primary Care Health Sciences<br>Sheppard, James; University of Oxford, Nuffield Department of Primary Care Health Sciences<br>Verbakel, Jan; KU Leuven (University of Leuven), Department of Primary Care; University of Oxford, Nuffield Department of Primary Care Health Sciences
**Primary Subject Heading**:	Pathology
Secondary Subject Heading:	Diagnostics, Evidence based practice
Keywords:	point of care testing, community pharmacy, Organisation of health services < HEALTH SERVICES ADMINISTRATION & MANAGEMENT, PRIMARY CARE

SCHOLARONE™ Manuscripts

For peer review only - http://bmjopen.bmj.com/site/about/guidelines.xhtml

I, the Submitting Author has the right to grant and does grant on behalf of all authors of the Work (as defined in the below author licence), an exclusive licence and/or a non-exclusive licence for contributions from authors who are: i) UK Crown employees; ii) where BMJ has agreed a CC-BY licence shall apply, and/or iii) in accordance with the terms applicable for US Federal Government officers or employees acting as part of their official duties; on a worldwide, perpetual, irrevocable, royalty-free basis to BMJ Publishing Group Ltd ("BMJ") its licensees and where the relevant Journal is co-owned by BMJ to the co-owners of the Journal, to publish the Work in this journal and any other BMJ products and to exploit all rights, as set out in our licence.

The Submitting Author accepts and understands that any supply made under these terms is made by BMJ to the Submitting Author unless you are acting as an employee on behalf of your employer or a postgraduate student of an affiliated institution which is paying any applicable article publishing charge ("APC") for Open Access articles. Where the Submitting Author wishes to make the Work available on an Open Access basis (and intends to pay the relevant APC), the terms of reuse of such Open Access shall be governed by a Creative Commons licence – details of these licences and which Creative Commons licence will apply to this Work are set out in our licence referred to above.

Other than as permitted in any relevant BMJ Author's Self Archiving Policies, I confirm this Work has not been accepted for publication elsewhere, is not being considered for publication elsewhere and does not duplicate material already published. I confirm all authors consent to publication of this Work and authorise the granting of this licence.

For peer review only

Impact of point-of-care tests in community pharmacies: a systematic review and meta-analysis

Ali Albasri[1], Ann Van den Bruel[2], Gail Hayward[1], Richard J McManus[1], James P Sheppard[1], Jan Y Verbakel[1*]

[1]Nuffield Department of Primary Care Health Sciences, University of Oxford, Woodstock Road, OX26GG, Oxford, Oxfordshire, United Kingdom

[2]Academic Centre for Primary Care, KU Leuven, Kapucijnenvoer 33 blok J, 3000 Leuven, Belgium

Email addresses:

ali.albasri@phc.ox.ac.uk,ann.vandenbruel@kuleuven.be,gail.hayward@phc.ox.ac.uk, richard.mcmanus@phc.ox.ac.uk, james.sheppard@phc.ox.ac.uk, jan.verbakel@phc.ox.ac.uk

[*]Corresponding author: Jan Y Verbakel, Nuffield Department of Primary Care Health Sciences, Radcliffe Primary Care Building, Woodstock Road, Oxford OX2 6GG, UK jan.verbakel@phc.ox.ac.uk

Keywords

Point-of-care-testing, community pharmacies, randomized controlled trials, systematic review

Word count: 2,962

For peer review only - http://bmjopen.bmj.com/site/about/guidelines.xhtml

Abstract

Objectives

To summarise the literature regarding the use of POCT in pharmacies versus control/usual care.

Design and setting

Systematic review and random-effects meta-analysis in community pharmacy.

Registration

PROSPERO registration: CRD42017048578

Data sources

MEDLINE, Cochrane Central Register of Controlled Trials, EMBASE, ClinicalTrial.gov and Web of Science databases were searched.

Eligibility criteria

Articles were included if they: involved a POCT conducted by a community pharmacist, member of pharmacy staff or local equivalent; measured a clinically relevant outcome e.g. clinical parameter monitoring. No clinical condition or language limits were set.

Patient and public involvement

No patient involvement

Data extraction and synthesis

Data were independently extracted by two members of the review team to capture changes in clinical care that resulted from the use of the POCTs. The methodological quality of included studies was assessed, using the Cochrane Risk of Bias tool and Newcastle-Ottawa scale.

Results

Thirteen of the 1,584 articles found were included in the meta-analyses. Studies covered four therapeutic areas: targeted anti-malarial therapy (n=3 studies), HbA1c in diabetes (n=2 studies), lipid control (n=3 studies), and INR control in patients taking Warfarin (n=5 studies). POCT in pharmacies reduced the risk of receiving antimalarial treatment when not clinically

For peer review only - http://bmjopen.bmj.com/site/about/guidelines.xhtml

indicated (RR 0.34 95% CI 0.31-0.37). Lipid and HbA1c control appeared largely unaffected by pharmacy POCTs, and the impact on INR time-in-therapeutic-range was inconclusive.

Conclusions

Only 4/13 included studies used a gold-standard randomised controlled trial (RCT) design, limiting our ability to conclusively determine the clinical utility of POCT conducted in pharmacies. Further RCTs are needed, particularly in areas such as upper respiratory tract infections, which have gathered momentum among service commissioners in recent years.

Article summary - Strengths and limitations of this study

- This review provides a timely and comprehensive overview of the current evidence related to POCT in pharmacies
- The majority of included studies were observational and were generally of poor methodological quality
- Pooling of data from a small number of studies per comparison led to high levels of observed statistical heterogeneity across a majority of comparisons.
- The review places into context the need for evidence-based policy making regarding the use of POCT.

For peer review only - http://bmjopen.bmj.com/site/about/guidelines.xhtml

Introduction

A point-of-care test (POCT) can be defined as a test performed by a qualified member of staff nearby the patient, where results are made available within the same clinical visit to support clinical decision making.[1] These tests have the potential to save clinical time and improve patient access to care in the form of diagnoses, medications or dose amendments.[2]

Interest in the use of POCTs in different healthcare settings is increasing and is expected to grow significantly in the years to come.[3] In 2016, the 'Community pharmacy forward view' (CPFV) was published as a response by the pharmacy sector to the then 'NHS five year forward view', and suggested that diagnostics and POCTs should be made routinely available in pharmacy settings.[4]

Given the current strain on primary healthcare services,[5] the provision of POCTs has become more commonplace in UK community pharmacies, with particular emphasis on the potential for POCTs to aid both acute condition diagnosis and long-term condition management.[6] In 2016, NHS England approved a 'test and treat' service at a large pharmacy chain for patients presenting with sore throats, in an attempt to curb inappropriate antibiotic prescriptions and reduce burden on general practice.[7] However, the evidence behind the use of POCTs in pharmacies appears to be from either pilot studies, non-randomised studies, or studies with no comparator groups.[8] The evidence-base for implementing POCTs remains a concern more generally given that studies tend to focus on test performance (method comparison with central laboratory testing) rather than clinical or healthcare utilization outcomes.[9]

While previous work has focused on the analytical quality of POCTs used by community pharmacists,[10] this paper presents the findings of a systematic review and meta-analysis assessing the clinical impact of POCTs in community pharmacies on clinical outcomes and healthcare processes.

For peer review only - http://bmjopen.bmj.com/site/about/guidelines.xhtml

Methods

Search strategy

A comprehensive search strategy in MEDLINE, Cochrane Central Register of Controlled Trials, EMBASE, ClinicalTrial.gov and Web of Science was devised. An example of the MEDLINE search terms can be found in supplementary table 1 (online). Relevant articles from inception to 24/04/2019 were searched in addition to references of relevant reviews and articles that met our selection criteria. No language limits or study design filters were applied.

Selection of studies and inclusion criteria

Two members of the review team (AA and JYV) independently reviewed titles, abstracts and full texts. Studies screened by title, abstract and full text were eligible for inclusion if they met all of the following criteria:

1. A POCT conducted by a community pharmacist or member of community pharmacy staff (i.e. pharmacy technician, healthcare assistant, or local equivalent).
2. Clinically relevant outcome measures reported e.g. change in clinical care such as: referral, admission to hospital, morbidity, mortality, or rate of diagnosis, time in therapeutic range, duration of illness.
3. Patients of all ages presenting to a community pharmacy for any medical condition.

Randomised controlled trials, non-randomised but experimental and controlled studies including before-and-after and retrospective cohort studies were included in this review. Systematic reviews were excluded but their reference lists were searched for relevant primary studies.

Studies were excluded if any of the following criteria applied:

1. Were diagnostic accuracy studies (focusing only on the performance of one or more point-of-care tests versus a central lab test).
2. Included only hospital inpatients.
3. Studies without a control group or comparator.
4. Patients self-testing or tests that were taken away by patients (to test at home, for example).
5. Included a POCT as part of a wider intervention, such that the effect of the POCT alone could not be ascertained.

For peer review only - http://bmjopen.bmj.com/site/about/guidelines.xhtml

Outcomes measured

The primary outcome of this review was the impact of POCT on clinically relevant outcomes such as changes to treatment, disease marker monitoring, referrals, admissions to hospital, morbidity, mortality, time to diagnosis, time in therapeutic range, or duration of illness.

Data extraction

Data were independently extracted and verified by two members of the review team (AA and JYV). Data were extracted to capture changes in clinical care that resulted from the use of the POCTs. The following data were extracted from the primary studies where available: referral or admission to other healthcare providers, mortality, morbidity, time in therapeutic range, percentage of patients reaching therapeutic targets such as cholesterol and HbA1c, resulting medication recommendations, or appropriateness of medication recommendations.

Quality assessment

The methodological quality of included studies was assessed independently by two authors (AA and JYV). Randomised trials were assessed using the Cochrane Risk of Bias tool [11] and included analysis of randomisation, allocation concealment, comparison of baseline characteristics and blinding. For non-randomised but experimental and controlled studies, the Cochrane Risk of Bias tool for observational studies was used. Case-control studies were assessed using the Newcastle-Ottawa scale.[12]

Data synthesis

Meta-analyses were conducted separately for randomised controlled trials and non-randomised studies whenever three primary studies or more were available per prespecified analysis. Data were analysed using a random-effects model due to expected heterogeneity in study designs and populations.[13] Analyses were grouped according to the condition to which the POCT related. Data were combined using the Review Manager (RevMan) version 5 software. For outcomes where meta-analysis was not possible, results were described qualitatively.

Where statistical heterogeneity was detected, possible contributing factors such as the setting or operator, the patient population, and/or other methodological characteristics were investigated in sensitivity analyses where possible. Where data allowed, publication bias was assessed via Egger's test to check for small study effects.[14]

For peer review only - http://bmjopen.bmj.com/site/about/guidelines.xhtml

Data are presented as a proportion of each study population, means with standard deviation (SD) or 95% confidence intervals (CI) unless otherwise stated.

The study protocol was registered on PROSPERO: International Prospective Register of Systematic Reviews and can be found online (http://www.crd.york.ac.uk/prospero) – registration number [CRD42017048578].

Patient and public involvement

No patient involvement.

For peer review only

Results

Study selection

After removal of 619 duplicate records, 1,584 studies were identified from the literature searches and one additional record was found from citation searches. After title and abstract screening 1,513 studies were excluded leaving 71 studies to be screened by full text. Of these, 58 studies were excluded for the reasons stated in the PRISMA flow diagram (Figure 1) leaving 13 studies eligible for inclusion.

Study characteristics

Table 1 shows the characteristics of included studies: seven were observational (pre/post design),[15–21] four were randomised controlled trials (RCTs),[22–25], one a prospective controlled staggered parallel design study, and one a retrospective cohort study.[26] Studies were conducted in the USA (n=5), Canada (n=2), Australia (n=1), New Zealand (n=1), Ghana (n=1), Nigeria (n=1), India (n=1), Uganda (n=1) and included data from a total of 23,149 patients. All point of care tests were conducted by a community pharmacist(s) or local equivalent that received training in both delivering the POCT and in the subsequent treatment recommendation.

None of the RCTs or observational studies charged the patient directly for the POCT. Patients were most commonly recruited into the observational studies via clinician referral or through pharmacy list searches, with only one of the seven observational studies recruiting patients opportunistically. Three of the RCTs recruited patients opportunistically upon presentation to the pharmacy, with the fourth recruiting eligible patients by invitation from a clinical list.

Quality assessment

The overall methodological quality (supplementary Figures 1-3) was moderate across the five prospective controlled trials, with three studies exhibiting a high risk of detection bias (lack of blinding of the outcome assessors) and an unclear risk of reporting bias (no study protocol available).[22,24,27] The non-randomised and before-after studies generally did not provide sample size justifications, and four of these studies did not account for confounding variables in patient selection.[15,17,19,20] For the single case-control study, the comparability of cases and controls was scored as "high risk", due to significant differences in the selection procedure.[26]

For peer review only

For peer review only - http://bmjopen.bmj.com/site/about/guidelines.xhtml

Study author & year	Study design	Country	POC test (condition or level monitored)	Test conducted by	Location of POCT	Total population (n)	Mean (SD) or median age (IQR) (I/C), years	Gender (% M)
Ansah 2015	Cluster RCT	Ghana	Malaria testing (CareStart Malaria HRP2 (Pf), Apacor)	Chemical seller	Private drug retail shops/chemical shops	4,603	15 (6-29)/ 19 (6-32)	51%
Mbonye 2015	Cluster RCT	Uganda	Malaria testing (First Response Malaria Ag. Combo Rapid Diagnostic Test, Premier Med Corp)	Drug shop vender	"Drug shops"	15,517	NA	48.3%
Ikwuobe 2013	RCT	Nigeria	Malaria testing (SD Bioline Malaria Antigen Pf, Alere)	Pharmacist	Community pharmacy	1,226	30.8 (NA)	48.3%
Al Hamarneh 2013	Pre-post observational	Canada	Hba1c (DCA Vantage, Siemens)	Independent prescribing pharmacist	Community pharmacies	100	64 (10.4)	58%
Oyetayo 2011	Pre-post observational	USA	Hba1c (device not specified)	Pharmacist	Community pharmacy	126	NA	NA
Gerrald 2010	Pre-post observational	USA	Lipid profile testing (Cholestech LDX Analyzer, Alere)	Pharmacist	Outpatient clinic	81	64.9 (6.9)	79.1%
Peterson 2004	RCT	Australia	Total cholesterol (Accutrend GC, Roche Diagnostics)	Pharmacist	Pharmacist visiting at home	81	63.5 (12.1)/ 65.5 (11.0)	63%
Bluml 2000	Pre-post observational	USA	Lipid profile testing (Cholestech LDX Analyzer, Alere)	Pharmacist	Community Pharmacy	397	57 (NA)	48%
Deepalakshmi 2018	Prospective controlled parallel trial	India	INR (CoaguChek XS Plus, Roche Diagnostics)	Pharmacist	Community pharmacy	80	61.4 (3.1)	74.4%
Harrison 2015	Pre-post observational	New Zealand	INR (CoaguChek XS Plus, Roche Diagnostics)	Pharmacist	Community pharmacy	671	72 (13-97)	62.4%
Rossiter 2013	Pre-post observational	Canada	INR (CoaguCheck XS Machine, Roche Diagnostics)	Pharmacist	Pharmacist-led POC clinic	119	78.8 (NA)	48.7%
Wilson 2004	Pre-post observational	USA	INR (Coaguchek-S, Roche Diagnostics)	Pharmacist	Community pharmacy	19	61 (NA)	68%
Ernst 2003	Retrospective cohort	USA	INR (CoaguCheck (Boehringer Mannheim)	Pharmacist	Pharmacist led outpatient clinic	129	76.6 (12.8)	45%

Table 1: Baseline characteristics POC: point-of-care, SD: standard deviation, IQR: interquartile range, I/C: intervention group/control group, M: Male, INR: International Normalised Ratio, NA: Not Available, RCT: randomised controlled trial. Studies grouped according to point of care test used and chronologically within each test (most recent first)

For peer review only - http://bmjopen.bmj.com/site/about/guidelines.xhtml

Outcomes and Tests used

Malaria

Three randomized controlled trials (n= 20,699) investigated the use of POCT in the context of Malaria.[23–25] All three studies reported the difference in total use of anti-malarial drugs between POCT and usual care groups (Figure 2). Utilisation of POCT in a pharmacy setting reduced total anti-malarial use (risk ratio (RR) 0.58, 95% CI 0.54 to 0.62) over usual care, however pooled estimates exhibited significant statistical heterogeneity (I^2= 90%). For context, usual care most commonly consisted of pharmacists making decisions to supply anti-malarial drugs using their clinical judgement or other parameters such as the patient's temperature, without a rapid diagnostic test.

Two studies reported the difference between appropriately dispensed anti-malarial drugs (defined as: anti-malaria indicated, anti-malarial given) given to patients receiving POCT or usual care.[23,25] These trials found that the risk of receiving inappropriate antimalarial treatment was reduced in the pharmacy POCT group compared to usual care (RR 0.34 95% CI 0.31-0.37, I^2= 76%).

International Normalised ratio (INR)

Five studies (n= 1,018) investigated the use of POCT in the context of INR testing – four pre/post observational studies,[15–17,27] and one retrospective cohort study (Figure 3).[26] Pooled analysis of the pre/post observational studies showed no clear benefit for POCT in pharmacies for INR control, as measured by percentage of time in therapeutic range (TTR) of target INR.[28] Mean difference in percentage TTR between POCT and usual care group was 7.99% (95%CI -0.74% to 16.71%; I^2 = 99%) in favour of pharmacist POCT. The single retrospective cohort study found an increase of 19.90% (95% CI 12.45 to 27.35%) in favour of pharmacist POCT.

Lipids

Three studies investigated the use of POCT in pharmacies with regards to lipid monitoring. Two studies were pre-post observational studies,[20,21] and one was a randomised trial (Supplementary Figure 4).[22]

Total cholesterol (TC) was investigated in all three studies. The RCT showed no significant difference in TC levels over usual care at six months (mean difference -7.80, 95% CI -19.65 to

For peer review only - http://bmjopen.bmj.com/site/about/guidelines.xhtml

4.05mg/dL) whereas the pooled analysis of the two observational studies did suggest a significant decrease in TC between baseline and two year follow-up (mean difference -29.63mg/dL, 95% CI -35.29 to -23.98mg/dL; I^2 = 0%).

At two years, meta-analysis showed a significant decrease in LDL (low-density lipoprotein) cholesterol between POC and usual care groups (mean difference -28.90mg/dL, 95% CI -40.74 to -9.65mg/dL; I^2 = 70%). Furthermore, an increase in HDL (high-density lipoprotein) cholesterol was observed, however this was non-significant (mean difference 3.96mg/dL, 95% CI -0.80 to +8.72mg/dL; I^2 = 77%).

Mean TG (triglycerides), LDL and HDL cholesterol were measured in the two observational studies.[20,21] Mean TG concentration was reduced from baseline levels after two-year follow-up (mean difference -21.68, 95% CI -34.74 to -8.61mg/dL; I^2 = 0%).

HbA1c

Two observational studies (n=226) investigated the effect of POCT on HbA1c control among diabetic patients.[18,19] The studies did not find a significant difference between baseline and follow-up HbA1c measurements (-1.02%, 95% CI -2.59% to 0.54%; I^2 = 96%, Supplementary Figure 5).

For peer review only

For peer review only - http://bmjopen.bmj.com/site/about/guidelines.xhtml

DISCUSSION

Summary of findings

We identified 13 studies including over 23,000 patients evaluating the clinical impact of POCT based in pharmacies. The available evidence was generally of poor methodological quality, and only 4/13 studies were randomised controlled trials.

The findings of this review suggest that pharmacy-based POCT may be useful in guiding appropriate anti-malaria prescribing, particularly in low resource settings. Further use of POCT, such as in lipid control, appeared to show some promise although the limited number of studies meant this could not be confirmed and the practical application of these tests in practice were unclear. There was no evidence that the delivery of POCT alone improved INR time-in-therapeutic-range or HbA1c levels in the community pharmacy setting.

Strengths and limitations

This review provides a timely and comprehensive overview regarding the current evidence related to POCT in pharmacies. The search and review strategy meant that we were unlikely to have missed large numbers of eligible studies. However, studies where the use of POCTs were embedded amongst other interventions were not included in this review given the difficulty in isolating the effect of the POCT on the outcomes measured. Such studies may have also provide useful information regarding the contribution of POCT to clinical outcomes.

The majority of included studies were observational and were generally of poor methodological quality (supplementary Figures 1-3). Although this limits our understanding of the clinical benefits (or harms) of these POCTs delivered in pharmacies, it highlights a need for high quality primary studies in this area of clinical practice. Furthermore, the primary literature included in this review were of limited clinical scope, covering only four therapeutic areas (anti-malarial drugs, Hba1c, INR and lipid levels). There was no data on areas such as acute infections that commonly present to community pharmacies - something that NHS commissioners have considered introducing into community pharmacies in the UK.[7] There is therefore no strong evidence for the use of POCT for either chronic disease monitoring or acute disease diagnosis in the community pharmacy setting at present. In addition, none of the included studies were conducted in the UK, making the generalisability to UK primary care challenging.

For peer review only - http://bmjopen.bmj.com/site/about/guidelines.xhtml

A further limitation of this review concerned the pooling of data from a small number of studies per comparison, leading to high levels of statistical heterogeneity across a majority of comparisons. As a result, the data presented in this systematic review should be interpreted with caution, as the addition of further, larger, studies to this body of evidence are likely to influence these findings.

Comparison with previous literature

A systematic review published in 2018 by Buss et al. aimed to summarise the literature related to both analytical quality and effectiveness of POCT in pharmacies.[10] Unlike our review, Buss et al included studies where pharmacy POCT performance was compared to corresponding laboratory results. Our review included the two studies contained within Buss et al. that compared pharmacy POCT with a control.[15,17] In addition to these, we were able to include a further ten papers.

In 2016, the largest pharmacy chain in the UK conducted a single arm feasibility study in 35 pharmacies - offering POCT for group A streptococcal pharyngitis.[8] After CENTOR scoring, patients testing positive for group A streptococci were offered antibiotic treatment at the pharmacy. A total of 149/367 (40.6%) patients received a throat swab, and of these, 36/149 (24.2%) were positive for group A streptococci. Antibiotics were supplied to 9.8% (*n*=36/367) of patients accessing the service. The study concluded that it was feasible to deliver such a service. The study did not report any clinical outcomes and therefore was not included in this review, although the number of GP consultations prevented and the reduction in antibiotic use was estimated based on patient self-reporting. Our systematic review has demonstrated that, to date, the impact on both clinical outcomes and total healthcare utilisation are yet to be established with regards to acute bacterial infections from a pharmacy setting.

Implications for clinical practice

Policy makers have identified community pharmacies as appropriate locations for extended healthcare delivery.[29] This is due in part to the strain on other areas of the health system, the convenience offered to patients who can see a pharmacist without an appointment, and the fact that pharmacists are highly trained in the safe use of medications. However, this systematic review has highlighted that extending the role of pharmacists to delivering POCT may require further assessment before large-scale rollout. Furthermore, to provide POCT in the future,

For peer review only - http://bmjopen.bmj.com/site/about/guidelines.xhtml

pharmacists and their staff will require specific training on the tests they provide and in managing the results appropriately. Other considerations such as the practicality and safety associated with handling bodily fluids in pharmacies will also require resolution (e.g. a suitable location for patients to provide a urine sample), in addition to having an appropriate medico-legal framework to allow pharmacists to deliver such interventions. Additional considerations include the source of funding for such services (local, national or patient funded). The Strep A feasibility study mentioned above was paid for by patients.[8] How such a model would fit in with the current NHS is another consideration that may require extensive stakeholder deliberation, cost-effectiveness analyses and health inequalities assessment.

Furthermore, the application of the findings from the lipid control studies in this review may be limited, given that in the UK, lipids are most commonly managed on the basis of overall cardiovascular disease (CVD) risk scoring,[30] rather than a stand-alone clinic. Therefore, there is likely to be limited application of POCT for lipids alone, unless it is conducted as part of a CVD risk assessment in the pharmacy.

A policy document presented to the American Pharmacist Association policy committee in 2015-16 outlined the following as potential barriers to the uptake of POCT by pharmacists:[31]

1. Lack of payment mechanisms.
2. Lack of standardised training/education across the profession.
3. Lack of standardised documentation systems and follow-up procedures.
4. Inconsistency in providing POCT services (post-code lottery).
5. Perceived pushback from medical and other related health professionals.

In addition to the operational and practical barriers stated above, this review has highlighted that lack of evidence of effectiveness and healthcare utilisation may also be contributing factors to the lack of commissioning and uptake.

Conclusion and recommendations

The few studies available suggest some promise in the use of pharmacy-based POCT for appropriate anti-malarial dispensing in low-resource settings, and for the control of blood lipids – however even these results require cautious interpretation given the heterogeneity observed and lack of evidence on clinically relevant outcomes. This systematic review has identified gaps in the literature regarding the evidence for use of POCT in pharmacies, particularly in

For peer review only - http://bmjopen.bmj.com/site/about/guidelines.xhtml

areas such s the triage and treatment of common acute bacterial or viral respiratory tract infections, where no evidence was found.

Future studies could consider non-inferiority of clinical outcomes versus usual care if the intervention is shown to be safe and cost-effective. Other outcomes such as patient access to care and re-presentation to general practice/out of hours care should also be carefully recorded in future studies.

Policy recommendations for the introduction of POCT in pharmacies should be informed by well-conducted randomised controlled trials and economic analyses of each specific condition(s). Until such time as these data become available, caution is required before the widespread roll-out of POCT in pharmacies.

For peer review only

For peer review only - http://bmjopen.bmj.com/site/about/guidelines.xhtml

Figure legends:

Table 1: Baseline characteristics POC: point-of-care, SD: standard deviation, IQR: interquartile range, I/C: intervention group/control group, M: Male, INR: International Normalised Ratio, NA: Not Available, RCT: randomised controlled trial. Studies grouped according to point of care test used and chronologically within each test (most recent first)

Figure 1 – PRISMA flow diagram

Figure 2 – The effect of pharmacy point-of-care-testing on receiving anti-malarial treatment (top) and on the risk of receiving anti-malarial treatment when it was not clinically indicated (number of anti-malarial medications dispensed).

Figure 3 – The effect of pharmacy point-of-care-testing on International Normalised Ratio (INR) % time in therapeutic range (TTR).

Supplementary Table 1: Medline search strategy

Supplementary Figure 1 - Risk of bias for pre-post observational studies

Supplementary Figure 2 - Risk of bias for retrospective case-control study

Supplementary Figure 3 - Risk of bias for prospective controlled trials

Supplementary Figure 4 – The effect of pharmacy point-of-care-testing on lipid control - Total cholesterol, LDL cholesterol, HDL cholesterol, and Triglycerides (mg/dL)

Supplementary Figure 5 – The effect of pharmacy point-of-care-testing on glycated haemoglobin HbA1c control (%)

For peer review only - http://bmjopen.bmj.com/site/about/guidelines.xhtml

For peer review only

References:

1 Schols AMR, Dinant G-J, Hopstaken R, *et al.* International definition of a point-of-care test in family practice: a modified e-Delphi procedure. *Fam Pract* 2018;**35**:475–80. doi:10.1093/fampra/cmx134
2 Price CP, Kricka LJ. Improving healthcare accessibility through point-of-care technologies. In: *Clinical Chemistry*. 2007. 1665–75. doi:10.1373/clinchem.2006.084707
3 Abel G. Current status and future prospects of point-of-care testing around the globe. *Expert Rev Mol Diagn* 2015;**15**:853–5. doi:10.1586/14737159.2015.1060126
4 Pharmaceutical Services Negotiating Committee, Pharmacy Voice. Community Pharmacy Forward View. 2016.
5 Hobbs FDR, Bankhead C, Mukhtar T, *et al.* Clinical workload in UK primary care: a retrospective analysis of 100 million consultations in England, 2007–14. *Lancet* 2016;**387**:2323–30. doi:http://dx.doi.org/10.1016/S0140-6736(16)00620-6
6 Howick J, Cals JWL, Jones C, *et al.* Current and future use of point-of-care tests in primary care: an international survey in Australia, Belgium, The Netherlands, the UK and the USA. *BMJ Open* 2014;**4**. doi:10.1136/bmjopen-2014-005611
7 Lewis G. NHS England to roll out Boots sore throat scheme. *Chem Drug* 2016.
8 Thornley T, Marshall G, Howard P, *et al.* A feasibility service evaluation of screening and treatment of group A streptococcal pharyngitis in community pharmacies. *J Antimicrob Chemother* 2016;**71**:3293–9. doi:10.1093/jac/dkw264
9 McKenna C, Burch J, Suekarran S, *et al.* A systematic review and economic evaluation of the clinical effectiveness and cost-effectiveness of aldosterone antagonists for postmyocardial infarction heart failure. *Health Technol Assess (Rockv)* 2010;**14**. doi:10.3310/hta14240
10 Buss VH, Deeks LS, Shield A, *et al.* Analytical quality and effectiveness of point-of-care testing in community pharmacies: A systematic literature review. *Res Soc Adm Pharm* Published Online First: 20 July 2018. doi:10.1016/J.SAPHARM.2018.07.013
11 Higgins J, Green S. Cochrane Handbook for systematic reviews of interventions version. In: *The Cochrane Collaboration*. 2011. 2–3. doi:10.1002/9780470712184.ch5
12 GA Wells, B Shea, D O'Connell, J Peterson, V Welch, M Losos PT. The Newcastle-Ottawa Scale (NOS) for assessing the quality of non-randomized studies in meta-analyses. Ottawa Hosp. Res. Inst. 2013.http://www.ohri.ca/programs/clinical_epidemiology/oxford.asp
13 Higgins JP, Thompson SG, Spiegelhalter DJ. A re-evaluation of random-effects meta-analysis. *J R Stat Soc Ser A Stat Soc* 2009;**172**:137–59. doi:10.1111/j.1467-985X.2008.00552.x
14 Egger M, Davey Smith G, Schneider M, *et al.* Bias in meta-analysis detected by a simple, graphical test. *BMJ* 1997;**315**:629–34. doi:10.1136/BMJ.315.7109.629
15 Harrison J, Shaw JP, Harrison JE. Anticoagulation management by community pharmacists in New Zealand: an evaluation of a collaborative model in primary care. *Int J Pharm Pract* 2015;**23**:173–81. doi:10.1111/ijpp.12148
16 Rossiter J, Soor G, Telner D, *et al.* A Pharmacist-Led Point-of-Care INR Clinic: Optimizing Care in a Family Health Team Setting. *Int J Family Med* 2013;**2013**:691454. doi:10.1155/2013/691454
17 Wilson SJ-A, MacLellan E, Cox JL, *et al.* A Pilot Study Evaluating the Feasibility of Monitoring Oral Anticoagulant Therapy with Point-of-Care Testing in a Community Pharmacy. *Can J Hosp Pharm* 2004;**57**. doi:10.4212/cjhp.v57i3.369
18 Al Hamarneh YN, Charrois T, Lewanczuk R, *et al.* Pharmacist intervention for glycaemic control in the community (the RxING study). *BMJ Open* 2013;**3**:e003154.

For peer review only - http://bmjopen.bmj.com/site/about/guidelines.xhtml

doi:10.1136/bmjopen-2013-003154
19 Oyetayo OO, James C, Martinez A, *et al.* The Hispanic Diabetes Management Program: Impact of community pharmacists on clinical outcomes. *J Am Pharm Assoc* 2011;**51**:623–6. doi:10.1331/JAPhA.2011.09229
20 Bluml BM, McKenney JM, Cziraky MJ. Pharmaceutical care services and results in project ImPACT: hyperlipidemia. *J Am Pharm Assoc (Wash)*;**40**:157–65.
21 Gerrald KR, Dixon DL, Barnette DJ, *et al.* Evaluation of a pharmacist-managed lipid clinic that uses point-of-care lipid testing. *J Clin Lipidol* 2010;**4**:120–5. doi:10.1016/j.jacl.2010.02.001
22 Peterson GM, Fitzmaurice KD, Naunton M, *et al.* Impact of pharmacist-conducted home visits on the outcomes of lipid-lowering drug therapy. *J Clin Pharm Ther* 2004;**29**:23–30.
23 Ansah EK, Narh-Bana S, Affran-Bonful H, *et al.* The impact of providing rapid diagnostic malaria tests on fever management in the private retail sector in Ghana: a cluster randomized trial. *BMJ* 2015;**350**:h1019. doi:10.1136/bmj.h1019
24 Ikwuobe JO, Faragher BE, Alawode G, *et al.* The impact of rapid malaria diagnostic tests upon anti-malarial sales in community pharmacies in Gwagwalada, Nigeria. *Malar J* 2013;**12**:380. doi:10.1186/1475-2875-12-380
25 Mbonye AK, Magnussen P, Lal S, *et al.* A Cluster Randomised Trial Introducing Rapid Diagnostic Tests into Registered Drug Shops in Uganda: Impact on Appropriate Treatment of Malaria. *PLoS One* 2015;**10**:e0129545. doi:10.1371/journal.pone.0129545
26 Ernst ME, Brandt KB. Evaluation of 4 Years of Clinical Pharmacist Anticoagulation Case Management in a Rural, Private Physician Office. *J Am Pharm Assoc* 2003;**43**:630–6. doi:10.1331/154434503322452274
27 Deepalakshmi M D, Joseph C, Jacob C, *et al.* Management of Warfarin Therapy: A New Point-Of-Care Monitoring Service by Community Pharmacists in India. *J Young Pharm* 2018;**10**:350–3. doi:10.5530/jyp.2018.10.77
28 Schmitt L, Speckman J, Ansell J. Quality Assessment of Anticoagulation Dose Management: Comparative Evaluation of Measures of Time-in-Therapeutic Range. Kluwer Academic Publishers 2003.
29 National Health Service. Five Year Forward View. 2014.
30 NICE. Cardiovascular disease : risk assessment and reduction , including lipid modification. *NICE Guidel* 2016;**1**:1–44.
31 Marsha Gilbreath. Point-of-Care Testing Background Paper Prepared for the 2015–2016 APhA Policy Committee. 2013.

For peer review only

For peer review only - http://bmjopen.bmj.com/site/about/guidelines.xhtml

Contribution statement

JYV and AA conceived the study. AA, JYV did data extraction. AA and JYV performed the analyses, which were discussed with AVdB, GH, RM, JS. JYV and AA drafted this report and AVdB, GH, RM, JS co-drafted and commented on the final version. All authors had full access to all of the data (including statistical reports and tables) in the study and take responsibility for the integrity of the data and the accuracy of the data analysis. JYV affirms that the manuscript is an honest, accurate, and transparent account of the study being reported; that no important aspects of the study have been omitted and will act as guarantor. All authors have read and approved the final manuscript.

Competing interests

All authors have completed the ICMJE uniform disclosure form at www.icmje.org/coi_disclosure.pdf and declare: no support from any organisation for the submitted work; no financial relationships with any organisations that might have an interest in the submitted work in the previous three years; no other relationships or activities that could appear to have influenced the submitted work.

Funding

JYV, GH, AVdB are supported through the National Institute for Health Research (NIHR) Community Healthcare MedTech and IVD Co-operative. AA receives funding from the NIHR SPCR. RJM and JS receive support from Oxford NIHR CLAHRC. JS also receives funding from the Wellcome Trust/Royal Society via a Sir Henry Dale Fellowship (ref: 211182/Z/18/Z) and the NIHR School for Primary Care Research. RJM is a NIHR Senior Investigator. The views expressed are those of the authors and not necessarily those of the National Health Service (NHS), the National Institute for Health Research (NIHR), or the Department of Health and Social Care.

Data sharing statement

All data for these analyses are included in the manuscript or online appendices. No additional data are available.

Acknowledgements

JYV had full access to all the data in the study and takes responsibility for the integrity of the data and the accuracy of the data analysis.

For peer review only - http://bmjopen.bmj.com/site/about/guidelines.xhtml

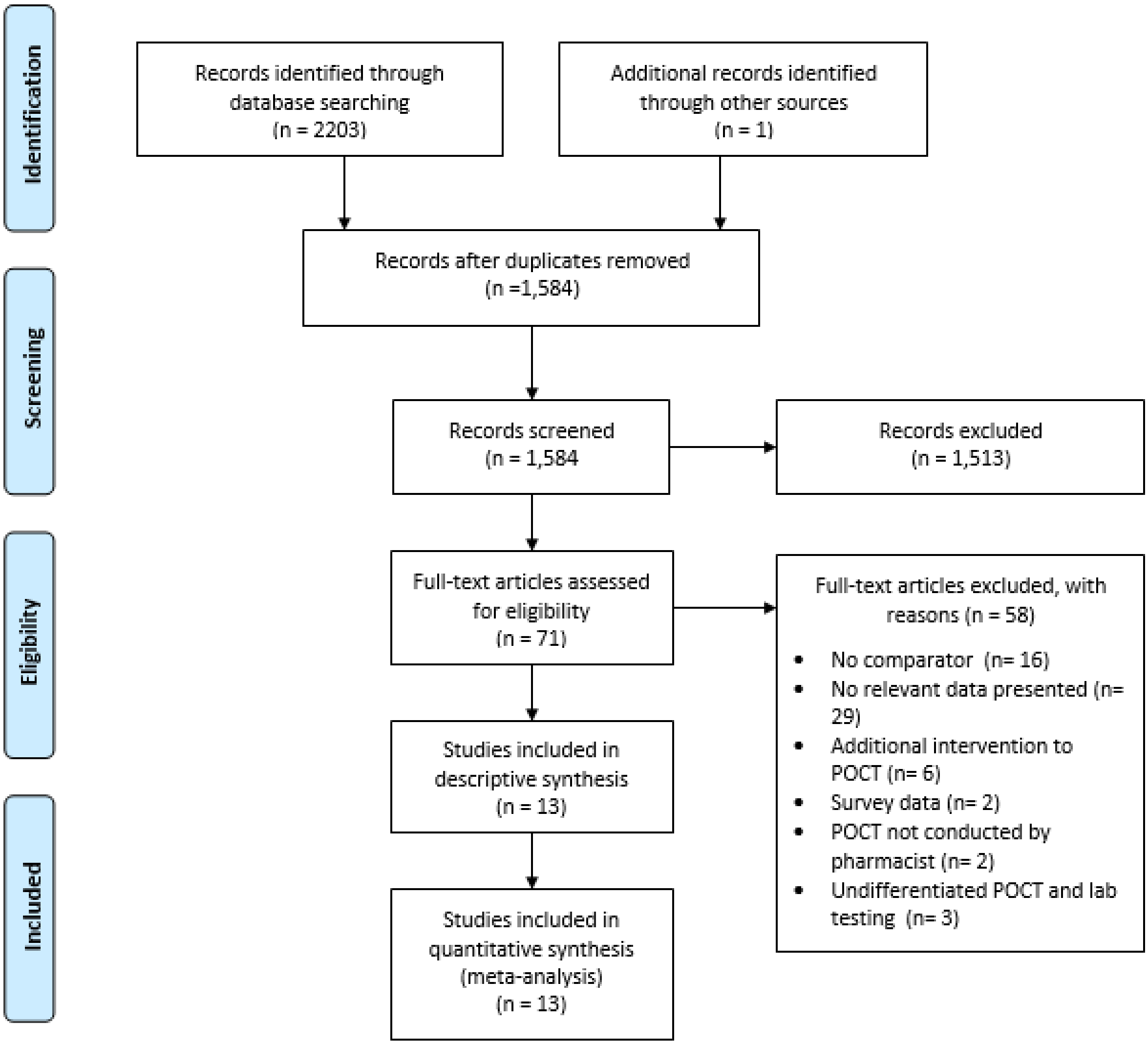

Figure 1 - PRISMA flow diagram

w only

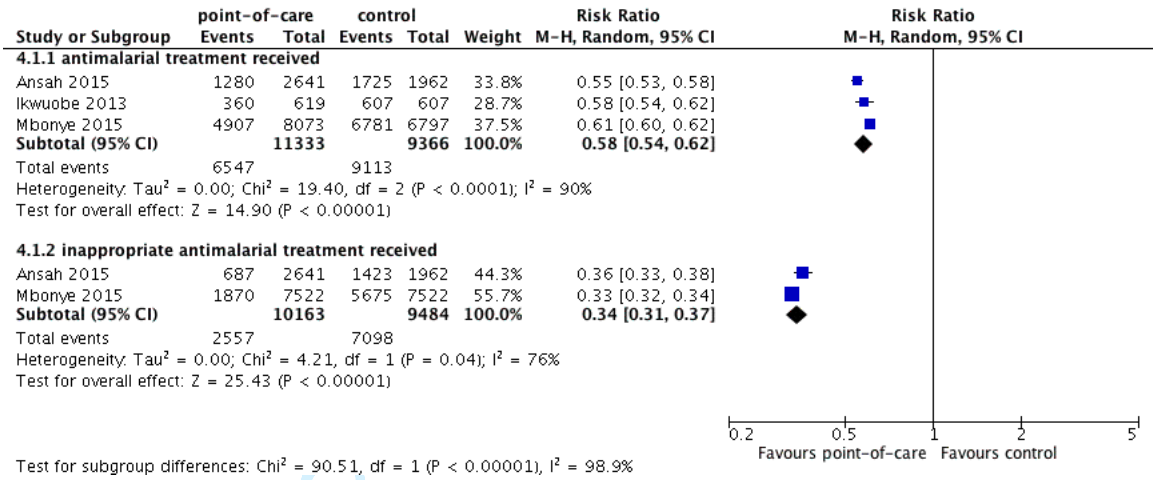

Figure 2 – The effect of pharmacy point-of-care-testing on receiving anti-malarial treatment (top) and on the risk of receiving anti-malarial treatment when it was not clinically indicated (number of anti-malarial medications dispensed).

For peer review only

Study or Subgroup	Point-of-care Mean [%]	Point-of-care SD [%]	Point-of-care Total	Usual care Mean [%]	Usual care SD [%]	Usual care Total	Weight	Mean Difference IV, Random, 95% CI [%]
**1.1.1 pre-post observational**								
Deepalakshmi 2018	87.3	4.7	44	77.5	15.8	36	30.8%	9.80 [4.46, 15.14]
Harrison 2015	80.3	8.85	671	78.6	3.35	671	34.7%	1.70 [0.98, 2.42]
Rossiter 2013	77.1	5.87	119	64.4	5.87	119	34.5%	12.70 [11.21, 14.19]
Wilson 2004	82	619	12	8	12.15	7		Not estimable
**Subtotal (95% CI)**			**834**			**826**	**100.0%**	**7.99 [−0.74, 16.71]**
Heterogeneity: $Tau^2 = 56.95$; $Chi^2 = 174.65$, df = 2 ($P < 0.00001$); $I^2 = 99\%$								
Test for overall effect: Z = 1.79 (P = 0.07)								
**1.1.2 retrospective cohort**								
Ernst 2003	57.5	12.8	80	37.6	22	39	100.0%	19.90 [12.45, 27.35]
**Subtotal (95% CI)**			**80**			**39**	**100.0%**	**19.90 [12.45, 27.35]**
Heterogeneity: Not applicable								
Test for overall effect: Z = 5.23 ($P < 0.00001$)								

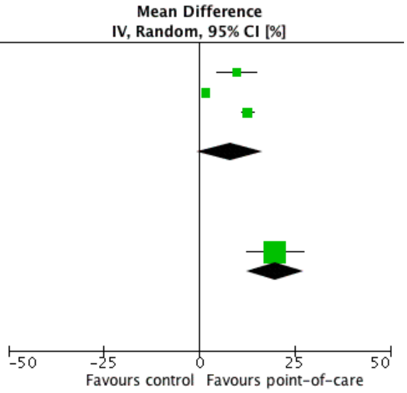

Figure 3 – The effect of pharmacy point-of-care-testing on International Normalised Ratio (INR) % time in therapeutic range (TTR).

For peer review only

# ▲	Searches
1	((rapid$ or same time or same visit or near patient or portable or handheld or hand-held) adj3 (test$ or analys$ or analyz$ or measure$ or assay$ or monitor* or device*)).ti,ab.
2	(fingerprick or finger prick).tw.
3	(poc or poct or "point of care").tw.
4	point-of-care systems/ or point-of-care testing/
5	1 or 2 or 3 or 4
6	Community Pharmacy Services/
7	Pharmacists/
8	(pharmacy or pharmacies or pharmacist?).ti,ab.
9	6 or 7 or 8
10	5 and 9
11	randomized controlled trial.pt.
12	controlled clinical trial.pt.
13	randomized.ab.
14	placebo.ab.
15	drug therapy.fs.
16	randomly.ab.
17	trial.ab.
18	groups.ab.
19	11 or 12 or 13 or 14 or 15 or 16 or 17 or 18
20	(animals not (humans and animals)).sh.
21	19 not 20
22	10 and 21
23	10 not 22

Supplementary Table 1: Medline search strategy

For peer review only

Supplementary Figure 1 - Risk of bias for pre-post observational studies

	study population defined	participation rate >50%	recruitment from same population	sample size justification	exposures measured prior to outcomes	reasonable timeframe between exposure and outcome	different levels of exposure examined	exposure measures clearly defined	exposure assessed more than once	outcome measure clearly defined	loss to follow up >20%	confounding variables measured
Al Hamarneh 2013	+	+	+	+	+	+	+	+	+	+	+	?
Bluml 2000	+	+	−	−	+	+	?	+	+	+	+	−
Gerrald 2010	+	+	+	−	+	+	?	+	?	+	+	+
Harrison 2015	+	+	+	−	+	+	?	+	+	+	+	−
Oyetayo 2011	+	?	?	−	+	+	?	?	+	+	?	−
Rossiter 2013	+	+	−	−	−	+	?	+	−	+	+	+
Wilson 2004	+	−	+	−	+	+	?	?	+	+	+	−

Supplementary Figure 2 - Risk of bias for retrospective case-control study

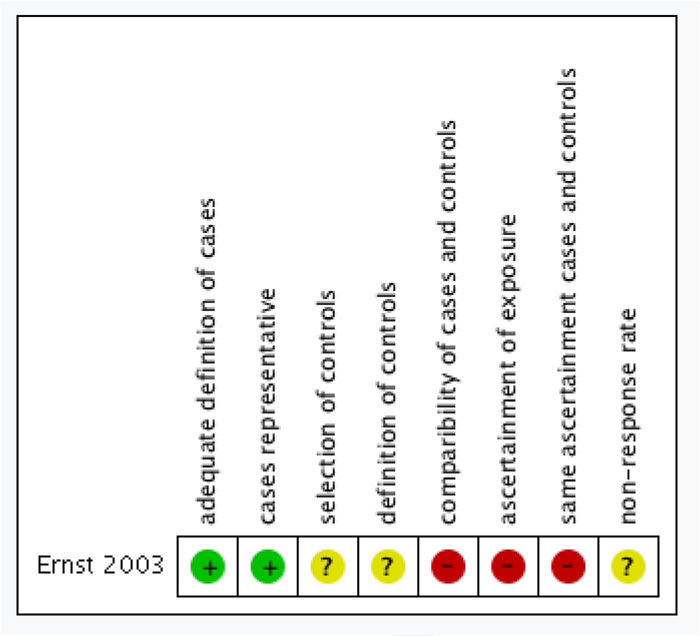

For peer review only

Supplementary Figure 3 - Risk of bias for prospective controlled trials

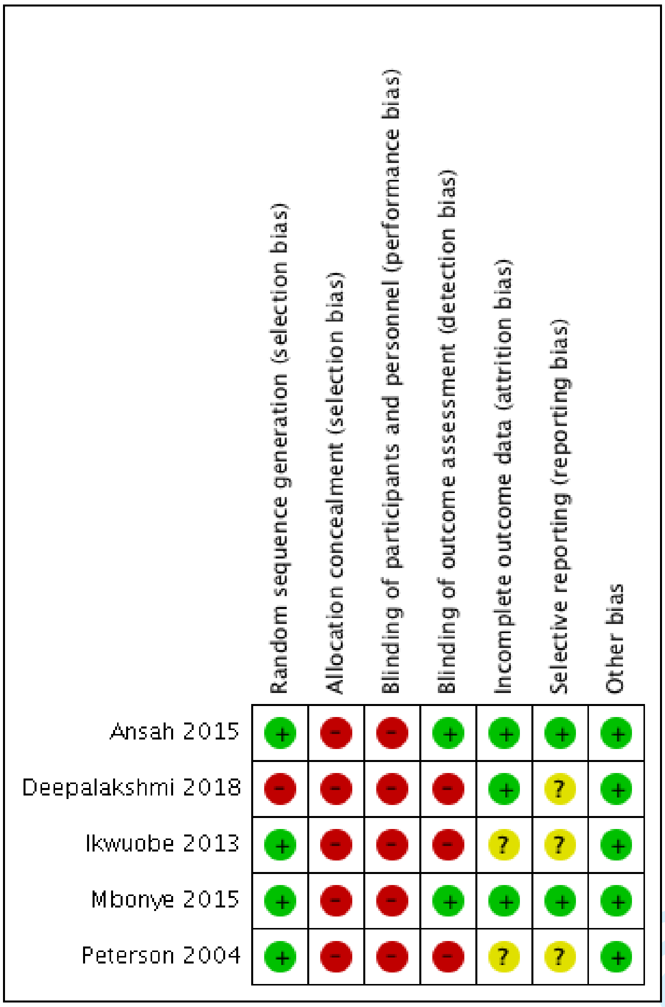

Study or Subgroup	point-of-care Mean [mg/dL]	point-of-care SD [mg/dL]	point-of-care Total	control Mean [mg/dL]	control SD [mg/dL]	control Total	Weight	Mean Difference IV, Random, 95% CI [mg/dL]
**3.5.1 total cholesterol randomised controlled trial**								
Peterson 2004	170.1	23.2	39	177.9	30.9	42	100.0%	-7.80 [-19.65, 4.05]
**Subtotal (95% CI)**			**39**			**42**	**100.0%**	**-7.80 [-19.65, 4.05]**
Heterogeneity: Not applicable								
Test for overall effect: Z = 1.29 (P = 0.20)								
**3.5.3 total cholesterol pre-post observational**								
Bluml 2000	207.5	41.1	396	238	46.7	396	85.1%	-30.50 [-36.63, -24.37]
Gerrald 2010	150.2	34.6	81	174.9	57.6	81	14.9%	-24.70 [-39.33, -10.07]
**Subtotal (95% CI)**			**477**			**477**	**100.0%**	**-29.63 [-35.29, -23.98]**
Heterogeneity: $Tau^2 = 0.00$; $Chi^2 = 0.51$, df = 1 (P = 0.47); $I^2 = 0\%$								
Test for overall effect: Z = 10.28 (P < 0.00001)								
**3.5.4 LDL-cholesterol**								
Bluml 2000	119.8	35.7	387	153.7	46.7	387	59.3%	-33.90 [-39.76, -28.04]
Gerrald 2010	81.5	28.1	76	103.1	45.1	76	40.7%	-21.60 [-33.55, -9.65]
**Subtotal (95% CI)**			**463**			**463**	**100.0%**	**-28.90 [-40.74, -17.06]**
Heterogeneity: $Tau^2 = 52.60$; $Chi^2 = 3.28$, df = 1 (P = 0.07); $I^2 = 70\%$								
Test for overall effect: Z = 4.78 (P < 0.00001)								
**3.5.5 HDL-cholesterol**								
Bluml 2000	49.2	16.5	394	43.1	14.1	394	56.3%	6.10 [3.96, 8.24]
Gerrald 2010	39.6	13.1	81	38.4	13.1	81	43.7%	1.20 [-2.83, 5.23]
**Subtotal (95% CI)**			**475**			**475**	**100.0%**	**3.96 [-0.80, 8.72]**
Heterogeneity: $Tau^2 = 9.29$; $Chi^2 = 4.42$, df = 1 (P = 0.04); $I^2 = 77\%$								
Test for overall effect: Z = 1.63 (P = 0.10)								
**3.5.6 triglycerides**								
Bluml 2000	195	91.3	394	216.6	111.3	394	84.5%	-21.60 [-35.81, -7.39]
Gerrald 2010	141.4	84.8	81	163.5	126.7	81	15.5%	-22.10 [-55.30, 11.10]
**Subtotal (95% CI)**			**475**			**475**	**100.0%**	**-21.68 [-34.74, -8.61]**
Heterogeneity: $Tau^2 = 0.00$; $Chi^2 = 0.00$, df = 1 (P = 0.98); $I^2 = 0\%$								
Test for overall effect: Z = 3.25 (P = 0.001)								

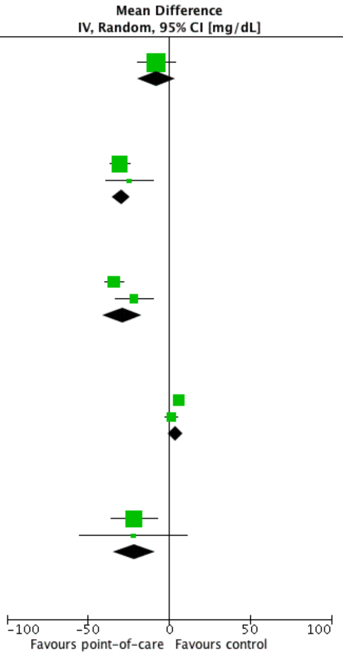

Test for subgroup differences: $Chi^2 = 90.94$, df = 4 (P < 0.00001), $I^2 = 95.6\%$

Supplementary Figure 4 – The effect of pharmacy point-of-care-testing on lipid control - Total cholesterol, LDL cholesterol, HDL cholesterol, and Triglycerides (mg/dL)

For peer review only

	point-of-care			control				Mean Difference
Study or Subgroup	Mean [%]	SD [%]	Total	Mean [%]	SD [%]	Total	Weight	IV, Random, 95% CI [%]
Al Hamarneh 2013	7.3	0.9	93	9.1	1	100	51.5%	−1.80 [−2.07, −1.53]
Oyetayo 2011	7.6	2.42	126	7.8	2.42	126	48.5%	−0.20 [−0.80, 0.40]
**Total (95% CI)**			**219**			**226**	**100.0%**	**−1.02 [−2.59, 0.54]**

Heterogeneity: $Tau^2 = 1.22$; $Chi^2 = 22.93$, df = 1 ($P < 0.00001$); $I^2 = 96\%$
Test for overall effect: Z = 1.28 (P = 0.20)

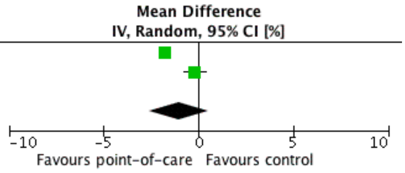

Supplementary Figure 5 – The effect of pharmacy point-of-care-testing on glycated haemoglobin HbA1c control (%)

For peer review only

PRISMA 2009 Checklist

Section/topic	#	Checklist item	Reported on page #
**TITLE**			
Title	1	Identify the report as a systematic review, meta-analysis, or both.	1
**ABSTRACT**			
Structured summary	2	Provide a structured summary including, as applicable: background; objectives; data sources; study eligibility criteria, participants, and interventions; study appraisal and synthesis methods; results; limitations; conclusions and implications of key findings; systematic review registration number.	2
**INTRODUCTION**			
Rationale	3	Describe the rationale for the review in the context of what is already known.	4
Objectives	4	Provide an explicit statement of questions being addressed with reference to participants, interventions, comparisons, outcomes, and study design (PICOS).	4,6
**METHODS**			
Protocol and registration	5	Indicate if a review protocol exists, if and where it can be accessed (e.g., Web address), and, if available, provide registration information including registration number.	7
Eligibility criteria	6	Specify study characteristics (e.g., PICOS, length of follow-up) and report characteristics (e.g., years considered, language, publication status) used as criteria for eligibility, giving rationale.	5
Information sources	7	Describe all information sources (e.g., databases with dates of coverage, contact with study authors to identify additional studies) in the search and date last searched.	5
Search	8	Present full electronic search strategy for at least one database, including any limits used, such that it could be repeated.	5
Study selection	9	State the process for selecting studies (i.e., screening, eligibility, included in systematic review, and, if applicable, included in the meta-analysis).	5
Data collection process	10	Describe method of data extraction from reports (e.g., piloted forms, independently, in duplicate) and any processes for obtaining and confirming data from investigators.	6
Data items	11	List and define all variables for which data were sought (e.g., PICOS, funding sources) and any assumptions and simplifications made.	6
Risk of bias in individual studies	12	Describe methods used for assessing risk of bias of individual studies (including specification of whether this was done at the study or outcome level), and how this information is to be used in any data synthesis.	6
Summary measures	13	State the principal summary measures (e.g., risk ratio, difference in means).	6
Synthesis of results	14	Describe the methods of handling data and combining results of studies, if done, including measures of consistency (e.g., I^2) for each meta-analysis.	6, 7

For peer review only - http://bmjopen.bmj.com/site/about/guidelines.xhtml

PRISMA 2009 Checklist

Section/topic	#	Checklist item	Reported on page #
Risk of bias across studies	15	Specify any assessment of risk of bias that may affect the cumulative evidence (e.g., publication bias, selective reporting within studies).	6
Additional analyses	16	Describe methods of additional analyses (e.g., sensitivity or subgroup analyses, meta-regression), if done, indicating which were pre-specified.	6
**RESULTS**			
Study selection	17	Give numbers of studies screened, assessed for eligibility, and included in the review, with reasons for exclusions at each stage, ideally with a flow diagram.	8
Study characteristics	18	For each study, present characteristics for which data were extracted (e.g., study size, PICOS, follow-up period) and provide the citations.	8
Risk of bias within studies	19	Present data on risk of bias of each study and, if available, any outcome level assessment (see item 12).	8
Results of individual studies	20	For all outcomes considered (benefits or harms), present, for each study: (a) simple summary data for each intervention group (b) effect estimates and confidence intervals, ideally with a forest plot.	9
Synthesis of results	21	Present results of each meta-analysis done, including confidence intervals and measures of consistency.	9,10 table 1, figures
Risk of bias across studies	22	Present results of any assessment of risk of bias across studies (see Item 15).	Suppl fig 1
Additional analysis	23	Give results of additional analyses, if done (e.g., sensitivity or subgroup analyses, meta-regression [see Item 16]).	Supple fig 2-5
**DISCUSSION**			
Summary of evidence	24	Summarize the main findings including the strength of evidence for each main outcome; consider their relevance to key groups (e.g., healthcare providers, users, and policy makers).	11
Limitations	25	Discuss limitations at study and outcome level (e.g., risk of bias), and at review-level (e.g., incomplete retrieval of identified research, reporting bias).	11
Conclusions	26	Provide a general interpretation of the results in the context of other evidence, and implications for future research.	13,14
**FUNDING**			
Funding	27	Describe sources of funding for the systematic review and other support (e.g., supply of data); role of funders for the systematic review.	15

For peer review only

PRISMA 2009 Checklist

From: Moher D, Liberati A, Tetzlaff J, Altman DG, The PRISMA Group (2009). Preferred Reporting Items for Systematic Reviews and Meta-Analyses: The PRISMA Statement. PLoS Med 6(6): e1000097. doi:10.1371/journal.pmed1000097

For more information, visit: **www.prisma-statement.org**.

For peer review only

For peer review only - http://bmjopen.bmj.com/site/about/guidelines.xhtml
